# Combinatorial technology revitalized by DNA‐encoding

**DOI:** 10.1002/mco2.84

**Published:** 2021-08-23

**Authors:** Árpád Furka

**Affiliations:** ^1^ Department of Organic Chemistry Eötvös Loránd University Budapest Hungary

**Keywords:** combinatorial chemistry, DNA‐encoded combinatorial library, drug discovery, split and pool synthesis

## Abstract

Combinatorial chemistry invented nearly 40 years ago was welcomed with enthusiasm in the drug research community. The method offered access to a practically unlimited number of new compounds. The new compounds however are mixtures, and methods had to be developed for the identification of the bioactive components. This was one of the reasons why the method could not providethe expected cornucopia of new drugs. Among the different screening methods, two approaches seem to offer the best results. One of them is based on the intrinsic property of the combinatorial split and pool solid‐phase synthesis: One compound forms on each bead of the solid support. Different methods have been developed to encode the beads and identify the structure of compounds formed on them. The most important method applies DNA oligomers for encoding. As a second approach in screening, DNA‐encoded combinatorial libraries are synthesized omitting the solid support and the mixtures are screened in solution using affinity binding methods. Libraries containing billions and even trillions of components are synthesized and successfully tested, which led to the identification of a significant number of new leads.

## Graphical abstract

Combinatorial technology comprises the synthesis of a mixture containing a high number of compounds in one procedure and screening the mixture also in one procedure. The left part of the image shows a two‐step synthesis of a nine‐member library using three building blocks and three DNA codes in both steps. The central part shows screening the library by affinity‐based binding. The non‐binding molecules are washed away, and the code of the binding molecule is amplified then sequenced.

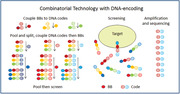

## 1 INTRODUCTION

Combinatorial Technology had been invented by the author of this article almost 40 years ago. The invention was described in a document notarized in 1982.^1^ Combinatorial technology is defined in the document as follows:

“The essence of the proposal is that instead of one by one synthesis of peptides, peptide mixtures should be prepared containing several hundred or several thousand peptides in approximately 1 to 1 molar ratio, and these peptide mixtures should be submitted to screening tests. It will be shown that on this way much labor can be saved both in the synthetic work and in the screening experiments.”

The copy of the full Hungarian document and the English version is a part of the Supplementary Materials. The basic idea of the invention is the use of compound mixtures in both synthesis and screening. The mixtures are so important that if a mixture is used in a synthesis, the procedure is inevitably combinatorial.

By replacing the single reactants used in conventional chemical syntheses with mixtures, millions and even billions of compounds can be easily synthesized. Both reactants can be replaced by mixtures. In the screening of mixtures, it is advantageous if the components of the library are present as close to equimolar quantities as possible. This was the reason for using only one mixture in the “split and pool” method^2^, ^3^, ^4^, ^5^ and choosing the solid phase in the stepwise synthesis of peptide libraries. In one cycle of the synthesis, the following operations are executed:

1. Split of the solid support into equal portions;
2. couple an amino acid to each portion;
3. pooling and thoroughly mixing the portions.

When a single reactant is coupled with a mixture of compounds, the number of newly formed compounds is equal to the number of components in the original mixture. As a consequence, the number of compounds increases exponentially with the number of coupling steps (cycles). This is the reason why combinatorial technology is so efficient. The best characterization of efficiency is the ratio of the number of the synthesized compounds to the number of couplings in the procedure. The ratio in conventional chemical synthesis is one or less, in the parallel synthesis, it is around a hundred, and in combinatorial synthesis, it is around millions.

The exceptionally high efficiency leads to enormous savings in pharmaceutical research. According to Goodnow's estimation,^6^ to create a collection of 1 million conventionally synthesized compounds and interrogate them by high‐throughput screening costs between $400 million and $2 billion, roughly $1100 per compound. A DNA‐encoded combinatorial library (DECL) of 800 million compounds, on the other hand, costs about $150,000 for materials to create and screen—approximately $0.0002 per compound.

In the beginning, peptide mixtures were synthesized. Since in the second operation of the cycles the coupling is done with only one amino acid, only one compound forms on each bead (see explanation in the Supplementary Materials: Formation of One Bead One Compound (OBOC) libraries).

The use of compound mixtures in the syntheses has a serious disadvantage: The structure of the produced compounds is unknown and finding a bioactive compound in a mixture of millions of peptides seemed to be similar to the task of finding a needle in a haystack. To overcome this difficulty, different deconvolution methods had been introduced.

The first such method is described in the 1982 document and can be used for screening of libraries cleaved from the beads of the support. The method was independently described by Erb et al. and named it “recursive deconvolution.”^7^ Another method developed for the same purpose is “positional scanning,” which uses pre‐prepared kits of sub‐libraries.^8^, ^9^ A method has also been introduced to determine the amino acid composition of bioactive peptides present in the solution of peptide libraries.^10^, ^11^ Although the use of these deconvolution methods is not widespread, they proved that “the needle can be found in the haystack.” Deconvolution methods are listed in the Supplementary Materials.

Another combinatorial method published by Fodor et al. does not need deconvolution. The library is synthesized on the surface of solid plates, and the position of compounds on the plate defines their identity.^12^

## 2 SCREENING AND ENCODING OBOC (One Bead One Compound) LIBRARIES

In the screening of OBOC libraries, the one‐bead‐one‐compound nature of the split‐and‐pool method is exploited. In one approach, the peptide libraries are incubated with a fluorescence dye‐labeled target protein, then the beads carrying the label are manually picked, and the peptides are sequenced by Edman degradation^13^ or tandem mass spectrometry.^14^ Later on, flow cytometers were applied for automatic sorting of the fluorescent beads.^15^, ^16^

Cha et al. automated the process from synthesis to screening. They applied Titan 357 bought from aapptec for automatic synthesis and used automatic MS (Mass Spectometry) and tandem mass spectrometry for data acquisition.^17^ In another approach, the beads of OBOC libraries are automatically sorted into the wells of microtiter plates, and then their content is analyzed by high‐throughput screening (HTS) methods.^18^

The OBOC libraries have an advantage when compared to phage display libraries^19^: Unnatural amino acids and organic compounds can be used as building blocks (BBs), but in order to facilitate screening, encoding methods had been introduced. Two approaches are used. In the microparticle matrix encoding of beads of Meldal and Christensen, all beads are individually encoded by random inclusion of optical micro‐particles into the beads and their relative 3D (Three dimension) positions constitute the codes.^20^ Quantum dotes are also used for similar purposes.^21^ In these cases, before or after coupling with the BBs, the codes of all beads present in the portions have to be read and recorded.

In the other approach, the BBs and their coupling orders are encoded into the beads. Ohlmeyer et al. published a binary encoding method^22^ and applied it in the synthesis of peptide libraries. The encoding molecules are 18 halobenzenes carrying a varying length hydrocarbon chain that is attached to the beads through a cleavable spacer. The tagging molecules, after cleaving them from the beads are identified by electron capture gas chromatography. Grouping the 18 tagging molecules in a binary manner, they constitute 1,048,576 codes simple by their presence in the codes. Nikolajev et al. applied peptides sequences for encoding,^23^ and Shchepinov et al. used substituted trityl mass‐tags for the same purpose.^24^

Brenner and Lerner suggested the application of DNA oligomers for encoding in solid‐phase split and pool synthesis.^25^ As implementation of the method, a DNA encoded peptide library was synthesized using controlled‐pore glass beads as solid support.^26^ The last three decades proved the excellent applicability of the Brenner–Lerner method.

In synthesizing organic OBOC libraries, organic solvents are commonly used, while in building the DNA oligomers, an aqueous medium is needed.^27^ To solve the problem, Paegel et al. offered a method in which the steps of compound synthesis are executed in the organic solvent, while the parallel building up of the DNA‐encoding oligomers is carried out in an aqueous solution. The method is applied in the synthesis of a 75,645 member OBOC library using a linear scaffold with three diversity positions.^28^

Liu et al. introduced topologically segregated bifunctional beads, which are made by biphasic solvent strategy. The testing molecules are on the outer layer, while the coding tags occupy the interior of the beads and do not interfere with screening. The coding tags are peptides containing unnatural α‐amino acids.^29^

The OBOC libraries are easily synthesized, and good methods are available for screening. As a consequence, they are widely used in drug research. Dealing with individual beads in screening, however, slows the process and so it is less efficient than the use of DNA‐encoded soluble libraries discussed in Section 3.

## 3 DNA‐ENCODED SOLUBLE COMBINATORIAL LIBRARIES

Originally DNA‐encoding was proposed to encode the beads of OBOC libraries. In 2000, however, a significant innovation was described. Harbury and Halpin omitted the solid support in the synthesis of DNA‐encoded libraries and replaced it with the encoding DNA oligomers and ended up with soluble libraries.^30^ Using the solid phase split and pool synthesis, no more compounds can be synthesized than the number of beads in the solid support. This limitation was completely eliminated by Harbury and Halpin, but an important advantage was lost: the easy purification by filtering out the excess of reagents and the byproducts. Franzini et al. developed a purification method to solve the problem.^31^ The excess of DNA is biotinylated and captured on streptavidin‐coated sepharose.

Another gain of the soluble DNA‐encoded libraries was the possibility to apply solution‐phase affinity‐based binding experiments in screening. It was also very important for the introduction of next‐generation DNA sequencing that made it possible to determine the identity and quantity of all binding molecules in a single process and at a low cost.^32^ With these developments, DNA‐encoding practically revitalized the combinatorial technology of soluble libraries and made it one of the most important tools in pharmaceutical research.

A comparison of the most important operations performed in combinatorial technology and those of the DNA‐encoding procedures below clearly shows the novelty added by DNA‐encoding. The differences appear in bold.

Operations in combinatorial technology

1. Dividing the solid support into portions;
2. coupling a different BB to each portion;
3. mixing the portions;
4. repeating the 1–3 operations;
5. screening the final mixture.

Operations in the DNA‐encoded combinatorial technology

1. Dividing the solid support into portions;
2. coupling a different BB to each portion;
3. **coupling an encoding DNA oligomer to each portion;**
4. mixing the portions;
5. repeating the 1–4 operations;
6. screening the final mixture and **i** **dentifying the bioactive molecules by PCR (Polymerase chain reaction) amplification and sequencing**.

### 3.1 Types of DNA‐encoded soluble combinatorial libraries

A few years after the turn of the millennium, different types of DNA‐encoded soluble small molecule organic libraries were published.

#### 3.1.1 DNA‐templated synthesis (DTS) of encoded combinatorial libraries

**FIGURE 1:**
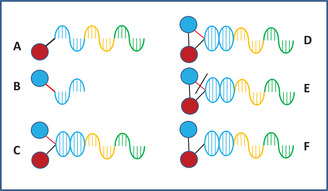
(A) Structure of a member of the template library with a building block (BB; red cycle) present in all members and three annealing regions (blue, yellow, and green). (B) A second library member with its code. (C) The second library member annealed to the template. (D) The bond formed between the two BBs. (E) Rupture of the cleavable bond. (F) Structure of the first cycle product

DTS^33^ is a DNA‐directed combinatorial process for making DECLs. It was developed in the laboratory of Prof. Liu at Harvard University. In the process, two libraries are used. One of them is the combinatorial template library. Members of the libraries are DNA strands with attached BB (red circle) at one end (Figure 1).

The DNA strand has annealing regions for attachment of the second, third, and so forth BBs. The second library contains a BB attached by a cleavable bond to the DNA coding region. When the two libraries are mixed, the coding region of the second BBs anneals with the annealing region on the strand on the template library. After annealing, the second BB is in proximity to the first one and can make a covalent bond with it. After cleaving the bond between the BB and its coding region, DECL is formed.

DTS allows the translation of libraries of DNA sequences into libraries of small molecules (peptide macrocycles) and offers the possibility to apply evolution‐based approaches. The DNA instruction sequences are mutated and reused during multiple rounds of translation, selection, and amplification to produce products with improved properties. O'Reilly et al. attempted to use organic BBs in DTS.^34^

#### 3.1.2 Synthesis of DECLs by sequence encoded routing

The synthesis based on sequence encoded routing is described by Halpin and Harbury^35^ It demonstrates a general method for the in‐vitro selection and evolution of combinatorial libraries.

**FIGURE 2:**
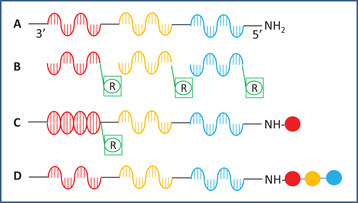
Synthesis of a trimer organic library by sequence encoded routing. (A) A member of the template library with the red, yellow, and blue coding regions for attachment of BBs. (B) Anticodons. (C) A member of the template library hybridized to an anticodon and coupled with the proper BB. (D) A member of the final DNA‐encoded library after coupling with the three BBs and eluted from the anticodon

In the synthesis of a small molecule trimer organic library, for example, DNA oligomers are prepared for encoding the BBs. Then from these oligomers using the split‐and‐pool method, a template library (library of genes) is constructed that directs the synthesis of the DECL. In the template library, there is a unique DNA‐encoding sequence for identifying every component of the encoded organic library sowing the identity of the BBs and their coupling order (Figure 2).

Resin‐bound anticodons are also prepared, one for each BB‐code. The anticodons of the first coupling positions are mixed one by one with the template library, and after hybridizing with the proper member of the library, they are separated by filtration from the rest of the library and then coupled with the proper BB. After finishing with the coupling of the BBs of the first coupling position, the library members are eluted, mixed, and the procedure is followed with the second, third, and so forth position anticodons and BBs. After the final round of hybridization, separation, coupling, and elution, the encoded library can be submitted to affinity‐based binding, PCR amplification, and sequencing.

The sequence encoded routing (also named directed chemical evolution) can also be used as an evolution‐based approach and to improve the product by multiple rounds of mutation, translation, selection, and amplification,^36^, ^37^

The sequence encoded routing and the DTS are elegant methods showing how natural evolution can be translated into a chemical system.

#### 3.1.3 DECLs formed by using a yoctoliter‐scale reactor

The use of yoctoliter‐size reactors for the synthesis of DECLs was described by Hansen et al.^38^ The combinatorial stepwise reactions take place between DNA‐encoded BBs confined into very small (yoctoliter‐size) space. Each BB is attached to a specially designed dsDNA oligomer that also contains the code of the BB. The BBs—except one of them—are attached by cleavable bonds. Two or three such DNA pieces can form at junctions of the yocto‐reactor. After the couplings of BBs take place and dismantling the reactor, the formed molecule remains attached to the oligonucleotide containing the encoding sequences of the BBs. The yocto‐reactor has the capability to perform molecular evolution by repeated rounds of mutation, translation, selection, and amplification.

#### 3.1.4 Dual pharmacophore DECLs

**FIGURE 3:**
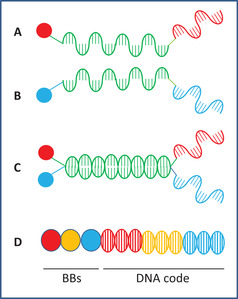
Self‐assembling (A, B, C) and single pharmacophore (D) DNA‐encoded combinatorial libraries (DECLs). (A) and (B) are the two sub‐libraries, BBs are color circles. The annealing regions are green, and the encoding regions are red and blue. (C) One member of the self‐assembled library. (D) Structure of the single pharmacophore DECL

The structure of a member of a dual‐pharmacophore combinatorial library is shown in Figure 3. Two sets of sub‐libraries are synthesized from two sets of BBs and two sets of partially complementary oligonucleotides, each containing a distinctive DNA barcode in Melkko et al.^39^ and Scheuermann and Neri.^40^

By mixing the two sets of sub‐libraries, the single set of the double‐stranded dual pharmacophore library forms by hybridization. If each of the two sub‐libraries contains 1000 components, mixing them in a combinatorial way leads to a 1,000,000 component final double‐stranded library. Each component of the library contains two BBs and the code of the two BBs. If both BBs are bound to the target protein, a synergetic effect may occur. Hits can be identified by affinity‐based binding.

#### 3.1.5 Single pharmacophore DECLs

In pharmaceutical research, the most often used DECLs are the single pharmacophore libraries that are constructed according to the combinatorial technology with an added modification. In the coupling cycles of the synthesis, in addition to coupling with the BBs, the DNA‐encoding oligomer is also elongated with the code of the BBs. The structure of a component of the library is demonstrated in Figure 3D.

Such a library was first described in a patent application of the Danish Nuevolution A/S entitled “Enzymatic encoding methods for efficient synthesis of large libraries*”* filed in December 2006 by nearly 30 inventors. Mannocci et al.^41^ published in 2008, a 4000 member library synthesized by the split‐and‐pool method from 20 amino acids and 200 carboxylic acids encoded by oligonucleotides. One year later, Clark et al.^42^ contributed to the field by synthesizing and screening a very large single pharmacophore library containing 800 million components.

Taking into account the Lipinski rule, usually small organic molecules are synthesized. If three sets of BBs are used in the synthesis, each set containing 1000 BBs, a 1 billion member library forms.

## 4 SCREENING

Screening involves affinity‐based methods, PCR amplification, sequencing of the encoding DNA oligomers, and evaluating the results.

In affinity‐based methods, the target proteins are immobilized on a solid support or can be used as solutions. It is very important that DNA‐encoding makes it possible to use both the library and the target protein in very low quantities. In a screening of the 4 billion member library of Deng et al. for example, the sum of the molar quantities of components in the affinity test was only 5 nmol. (around 100,000 molecules). The quantity of the target protein was also low at 10 μg.^43^

The members of the library are binding to the protein according to their affinity. The rest is washed away. The remaining part is submitted to PCR amplification and sequencing.

Sequencing of the encoding oligonucleotides of the components of the mixture is done in a single fast process. The result is not only the identity of the binding compounds but the sequence counts also contain information about their relative quantity. The results can be visualized by plotting the compounds in 3D space.

In the area of screening, an important new development was recently presented: screening of DECLs in living cells^44^ by injecting a 194 million member library into Xenopus laevis oocytes. The new method eliminates the need for highly purified active target proteins and performs the screening under physiologically relevant conditions. One of the identified leads appears as No. 1 in Table 1.

**TABLE 1:** Lead compounds identified from DNA‐encoded combinatorial libraries

**Num**.	**Structure**	**Library**	**Target**	**Ref**.
1	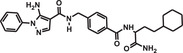	194 million	Xenopus laevis oocyte proteins	^44^
2	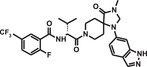	225 million	Autotaxin	^57^
3	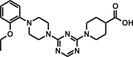	162 million	OXA‐48 Carbapenemase	^58^
4	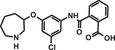	550 million	PqsE Thioesterase	^59^
5	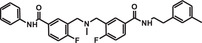	81 million	Bifunctional epoxide hydrolase 2	^60^
6	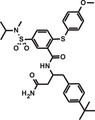	500 million	*β*_2_‐Adrenoceptor	^61^
7	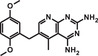	Billions	*Mycobacterium tuberculosis* DHFR (Dihydrofolate reductase)	^45^
8	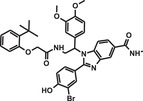	Billions	*Staphylococcus aureus* MRS (Methicillin‐resistant)	^45^
9	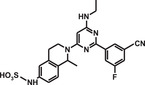	Billions	*Acinetobacter baumannii* LoIA (Outher‐membrane lipoprotein carrier protein)	^45^
10	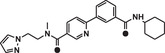	334 million	Soluble epoxide hydrolase	^62^
11	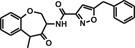	2 million	Receptor interacting protein 1 kinase	^63^
12	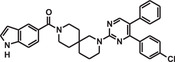	8 million	Hepatitis C virus NS4B protein	^64^

The automatic parallel screening applied by Machutta and his more than 60 co‐authors^45^ is also very important. They showed that hundreds of proteins can be screened in parallel. Antibacterial leads were identified against 119 targets from *Acinetobacter baumannii* and *Staphylococcus aureus* and additional leads by screening 42 targets from *Mycobacterium tuberculosis*. Examples are found in Table 1 (Nos. 7–9).

## 5 CONCLUSION

Combinatorial libraries as OBOC libraries and particularly in their DNA‐encoded form are extensively used in drug research. Pharmaceutical companies and academic research groups have already introduced combinatorial technology with DNA‐encoding in their drug discovery programs and are identifying more and more drug candidates. DNA‐encoding allows the full potential of combinatorial technology to be exploited to increase the number of new drugs. The costs of synthesis and screening are only small fractions of that of the conventional synthesis and HTS (High‐throughput screening)^6^ and allow simultaneous testing of millions of structurally related compounds providing SAR (structure activity relationships) databases and hits.^46^, ^47^, ^48^, ^49^ Reference 46 lists nearly 70 leads identified by different laboratories, and about half of them were published in the last 5 years; 12 lead structures appear in Table 1.

DNA‐encoding, however, has drawbacks, too. Many organic synthetic methods are excluded from the synthesis of DECL because the reaction conditions are incompatible with DNA. The newly reported DNA‐compatible reactions are expanding the usability of DNA‐encoding,^50^, ^51^, ^52^, ^53^, ^54^, ^55^ and machine learning can help to identify hits by analyzing the screening results.^56^

Another problem is that the encoding DNA oligomer is shading one part of the surface of the encoded compound. MacConnell et al. developed an off DNA‐encoded library screening method to solve the problem.^28^

To eliminate the shading property of the DNA‐encoding oligomers, the best would be to use combinatorial libraries without any encoding and find a method that can read the structure of the screened compounds that have an affinity to the targets without having any attached label. Of course, this seems impossible today, but what today is impossible in the future may be routinely used. The synthesis of billions of compounds seemed also impossible before 1982. So there is room for innovation.

The above‐mentioned two methods: screening in living cells and automatic parallel screening, promises a big leap in the speed and efficiency of pharmaceutical research, and it is expected to result in an increasing number of novel drugs to cure diseases in the near future. In addition, the automatic parallel screening helps to bring closer an old dream of the inventor of combinatorial technology: screening all combinatorial libraries against all available targets.

This short overview shows that combinatorial technology after nearly 40 years of its invention is still an eminent player in the drug discovery area.

## CONFLICT OF INTEREST

The author declares no conflict of interest.

## AUTHOR CONTRIBUTION

Árpád Furka wrote this paper and prepared the figures.

## ETHICS STATEMENT

Not applicable.

## Supporting information

Supporting Information

## Data Availability

Not applicable.
