# Supplementary material for: Combinatorial technology revitalized by DNA‐encoding
Source: MedComm (2020). 2021 Aug 23;2(3):481–9. doi: 10.1002/mco2.84 (PMC8554669; doi:10.1002/mco2.84)
Supplement: Supplementary file 1 — Supporting Information [file MCO2-2-481-s001.docx]

**Supporting Information**

to the article: Árpád Furka “Combinatorial Technology revitalized by DNA-encoding”

**The document notarized I 1982**

**A kombinatorikus kémia elveinek első megfogalmazása**

**The first description of the principles of combinatorial chemistry**


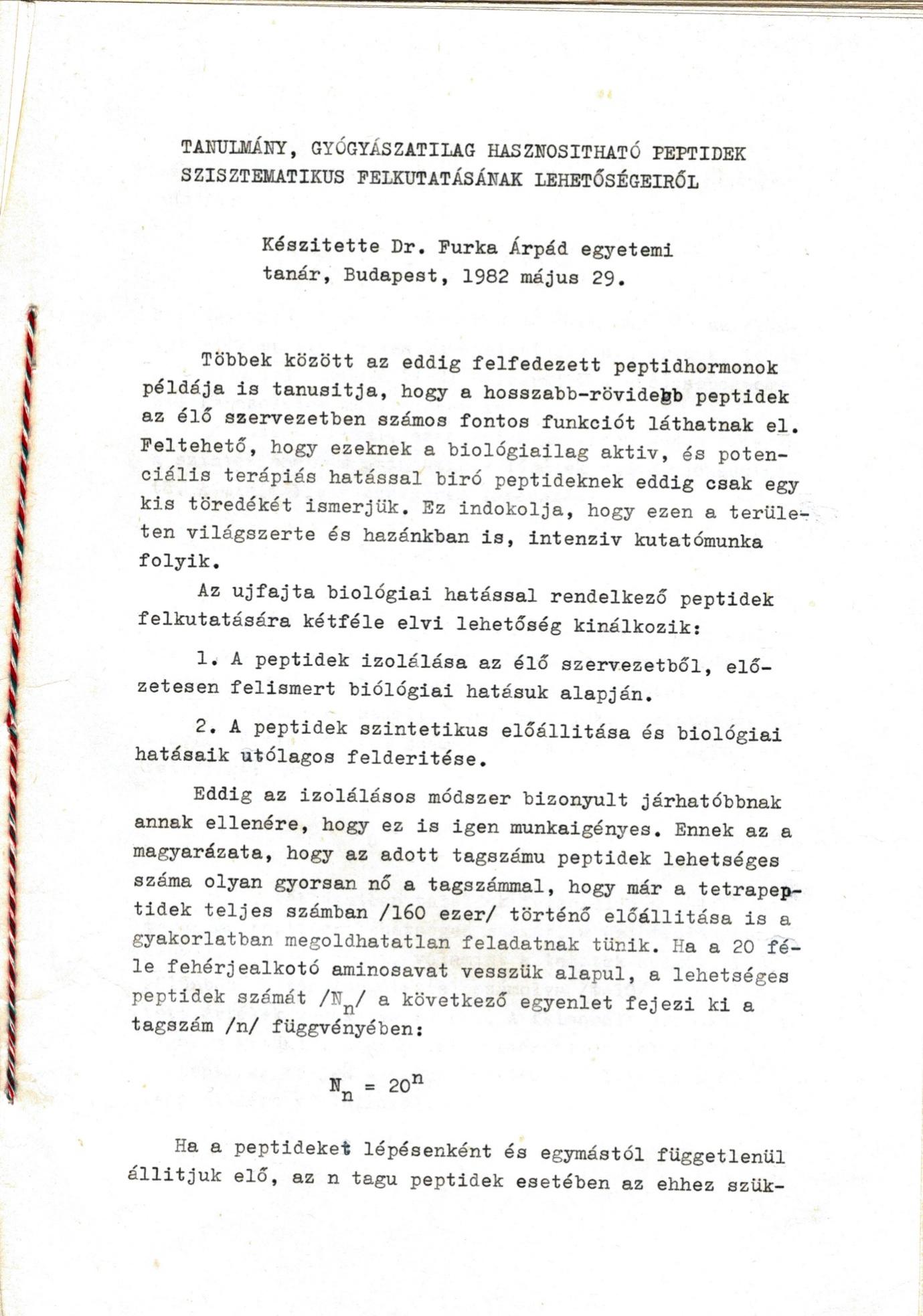


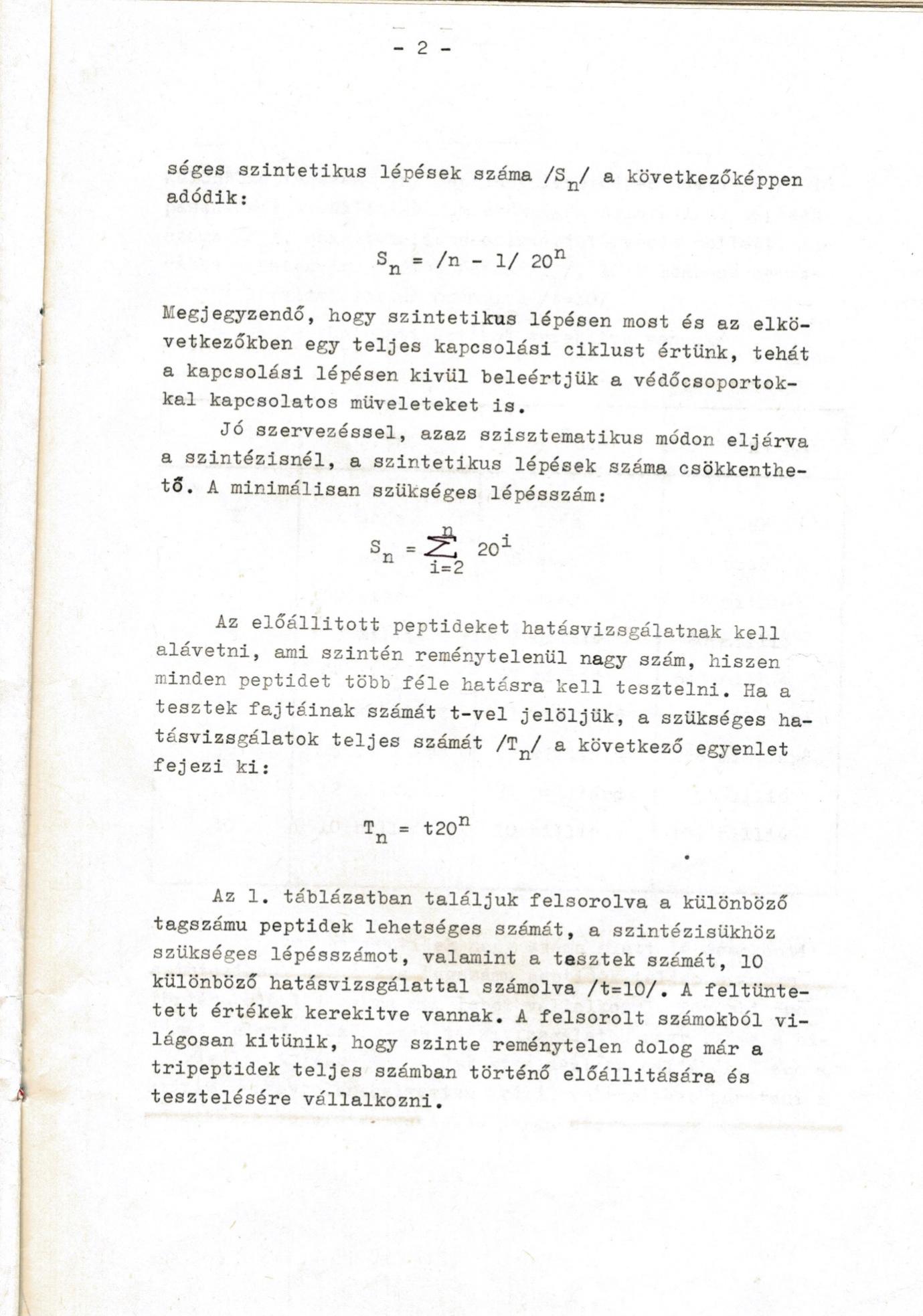


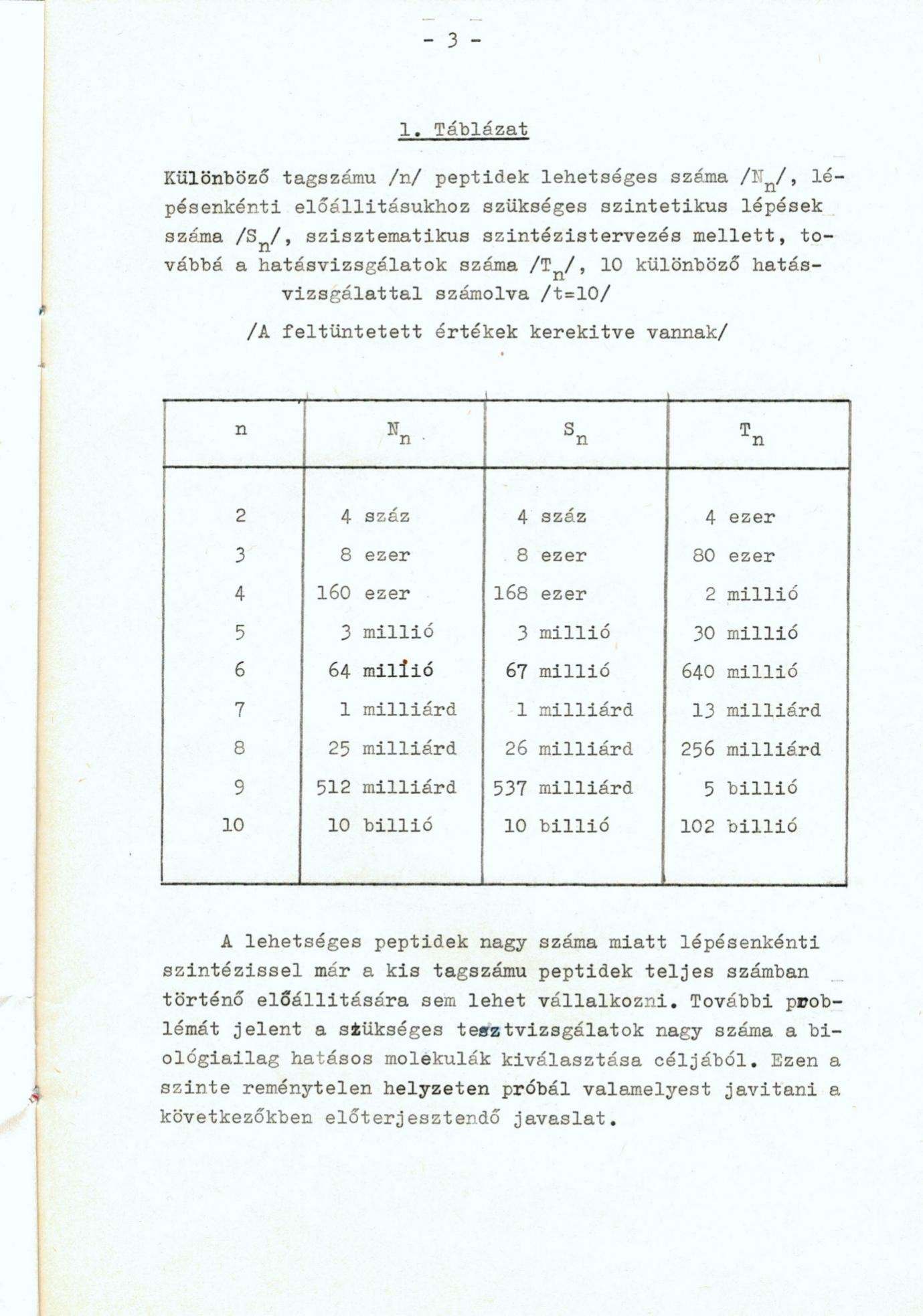


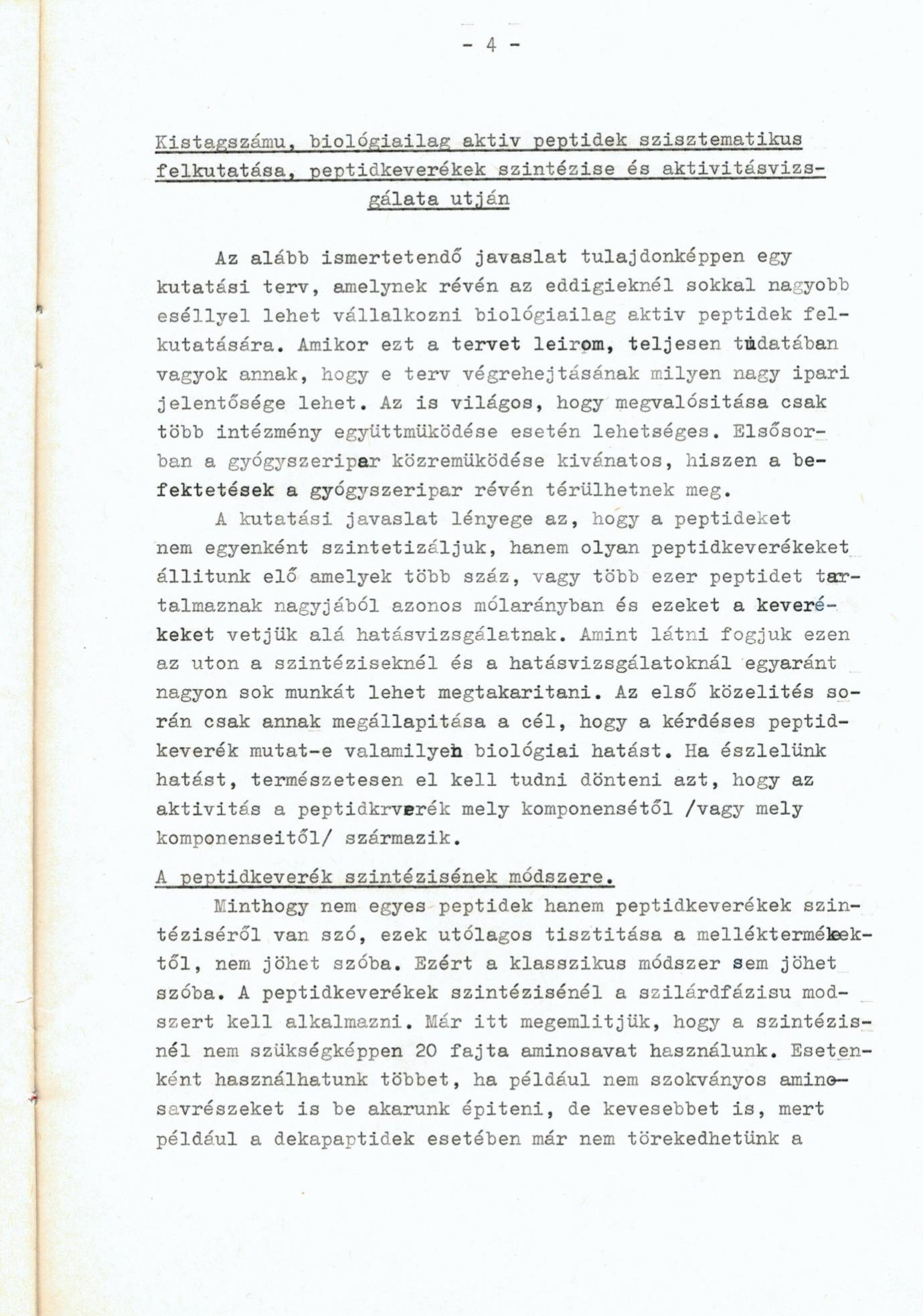


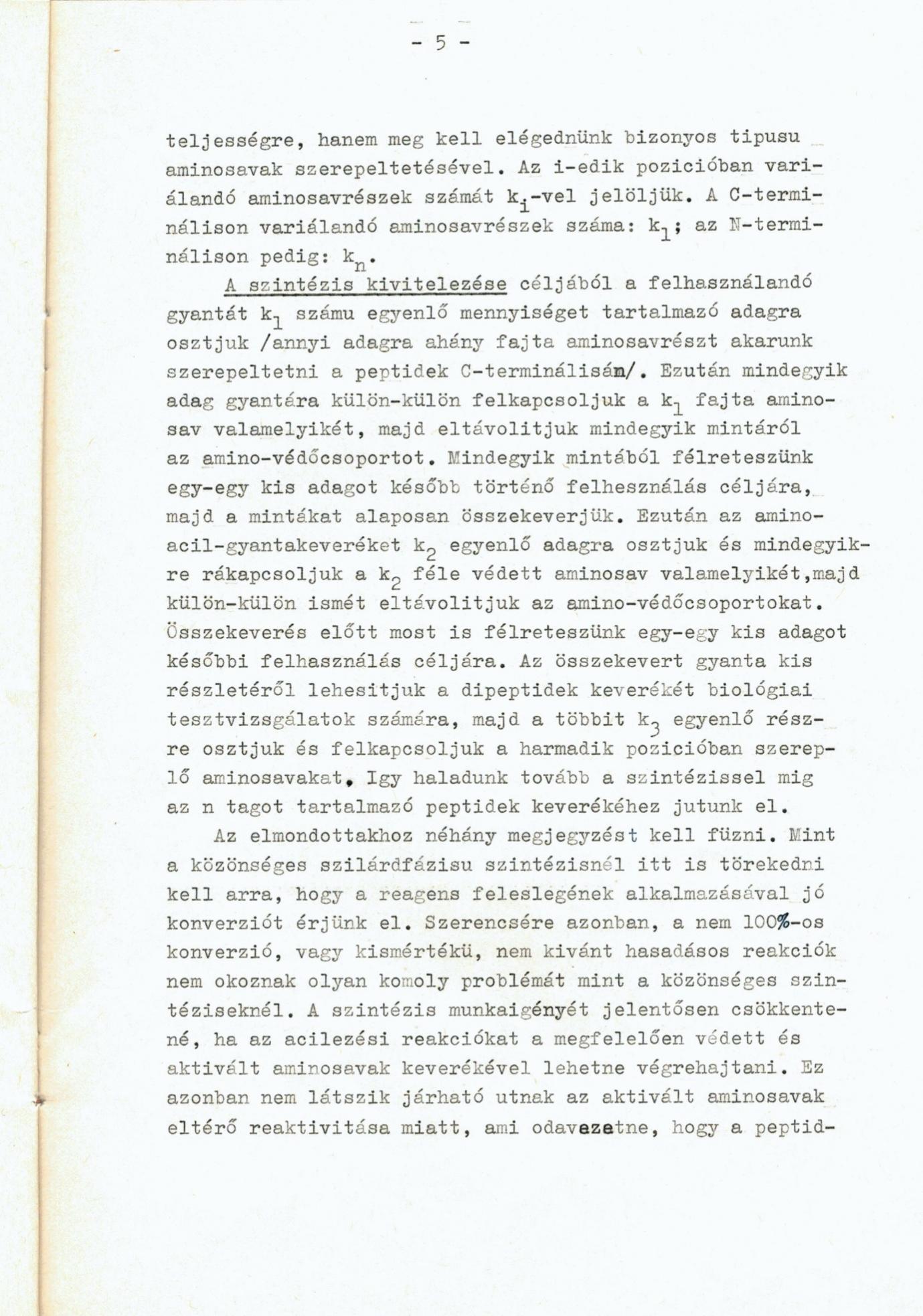


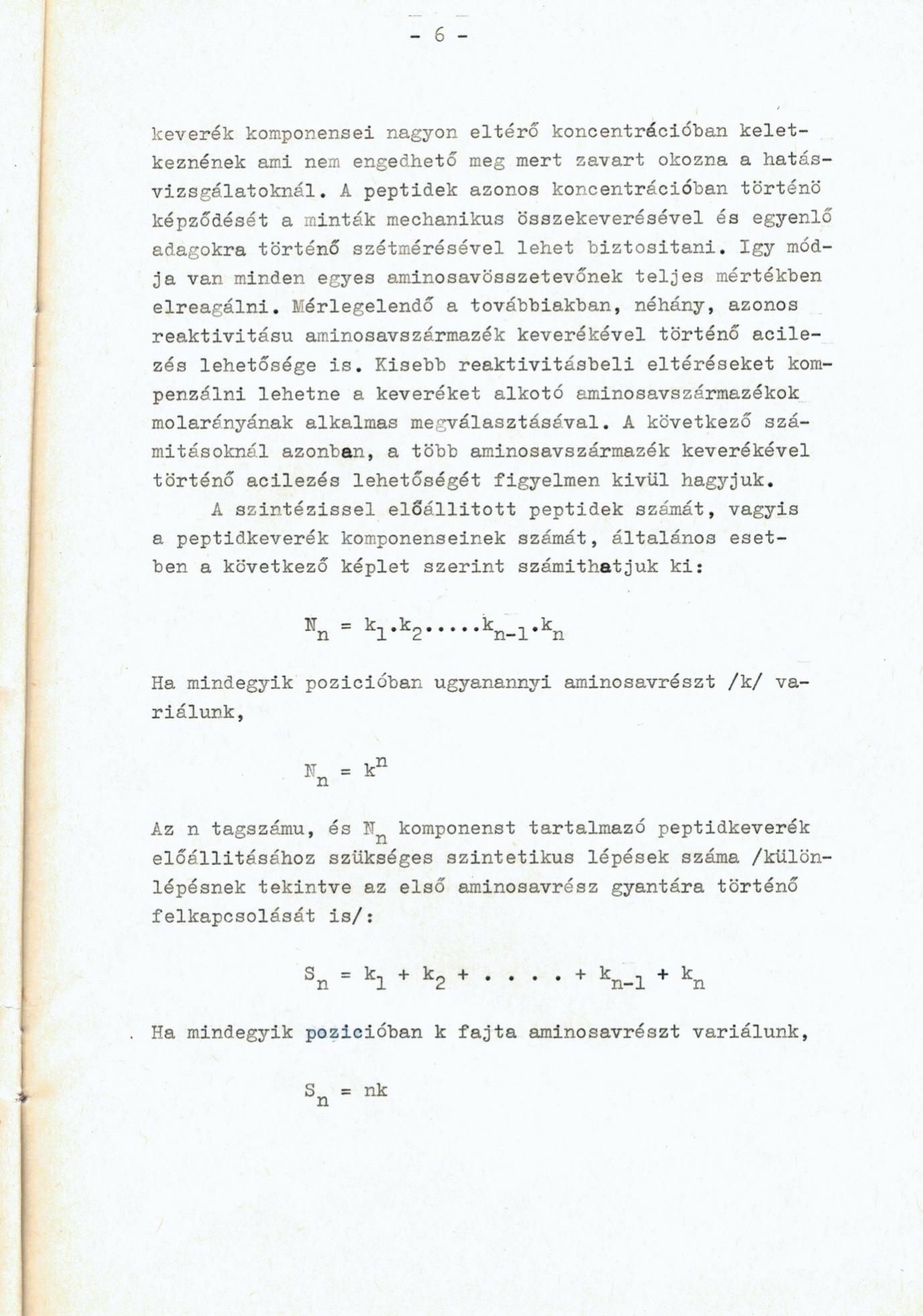


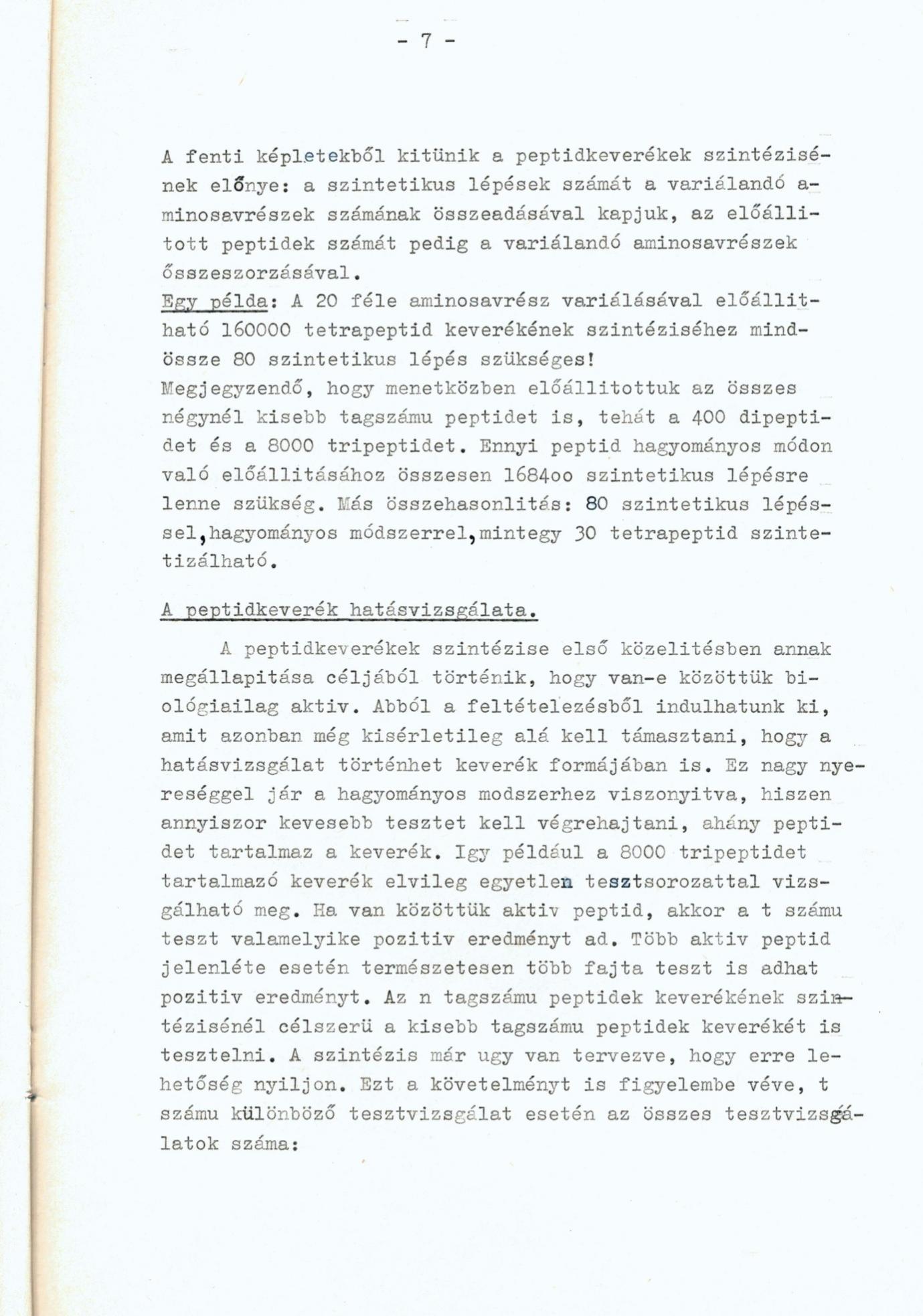


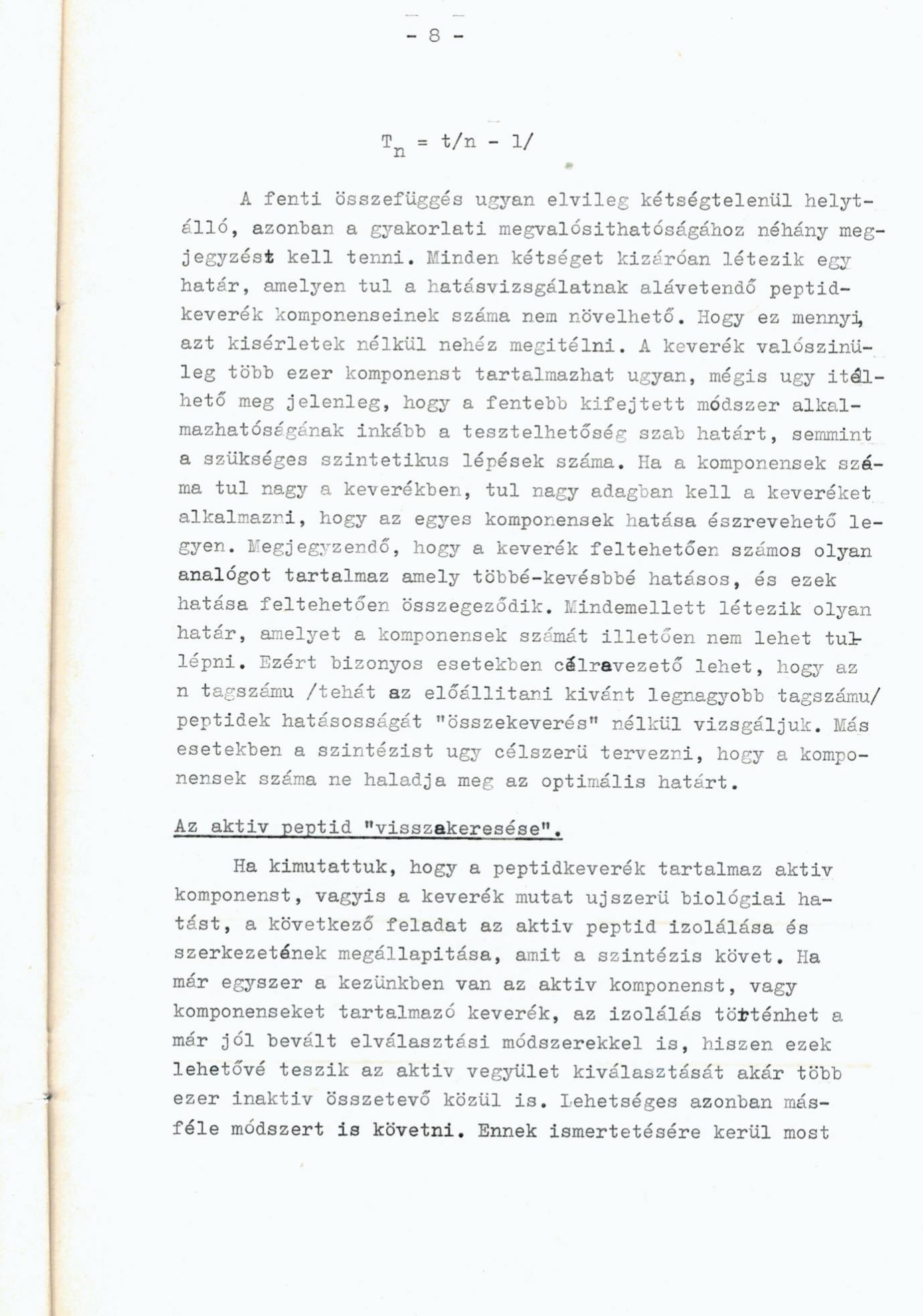


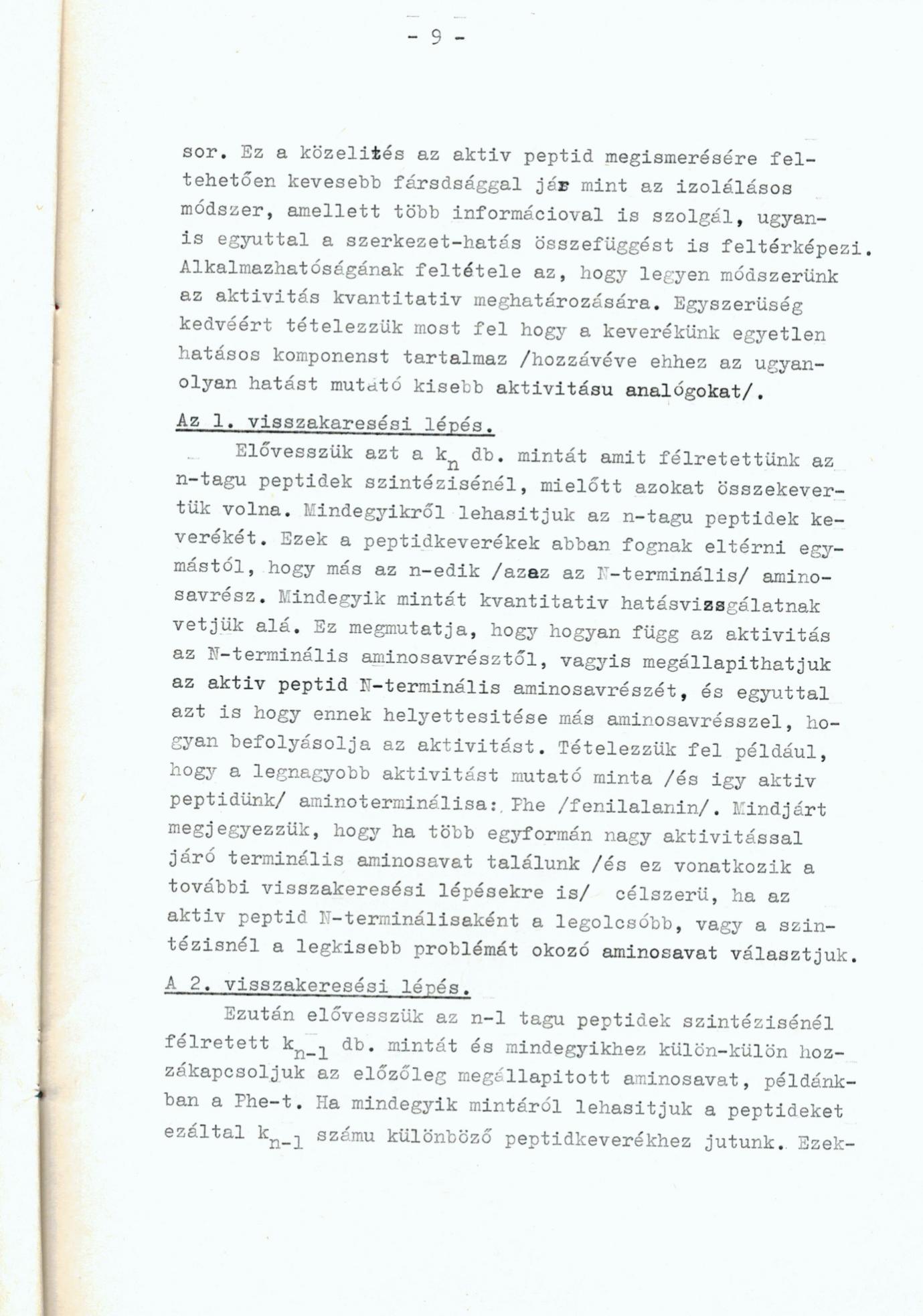


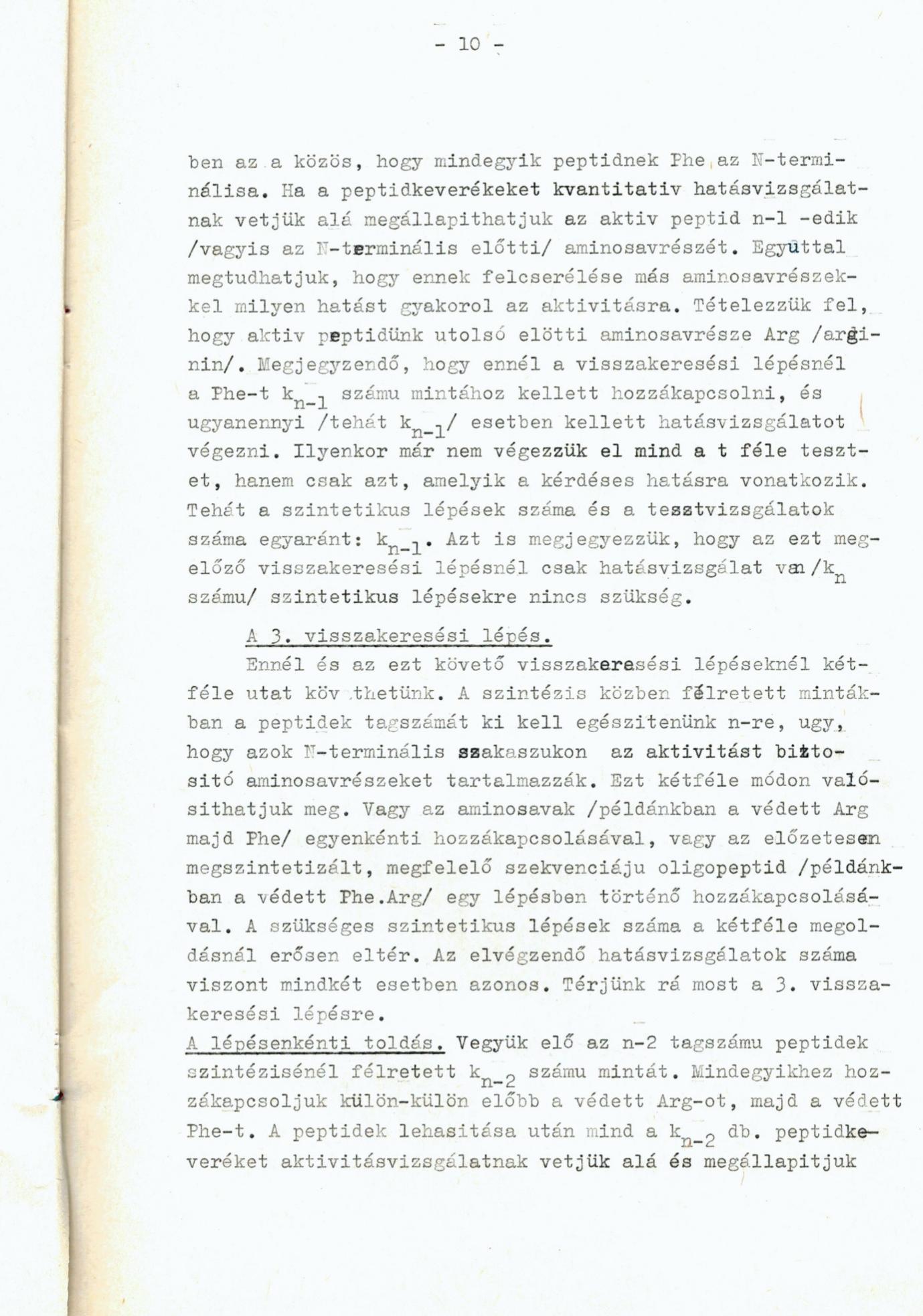


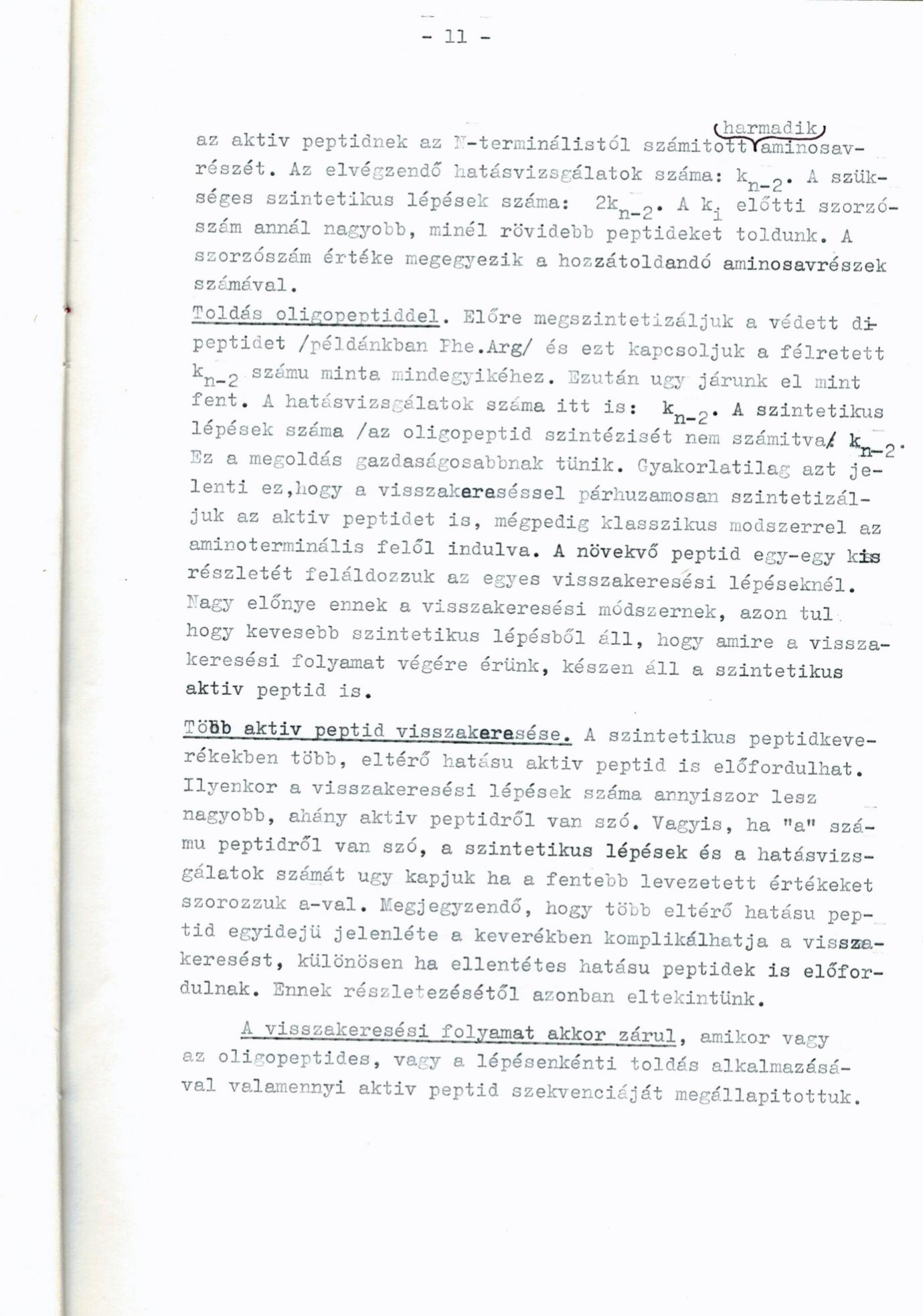


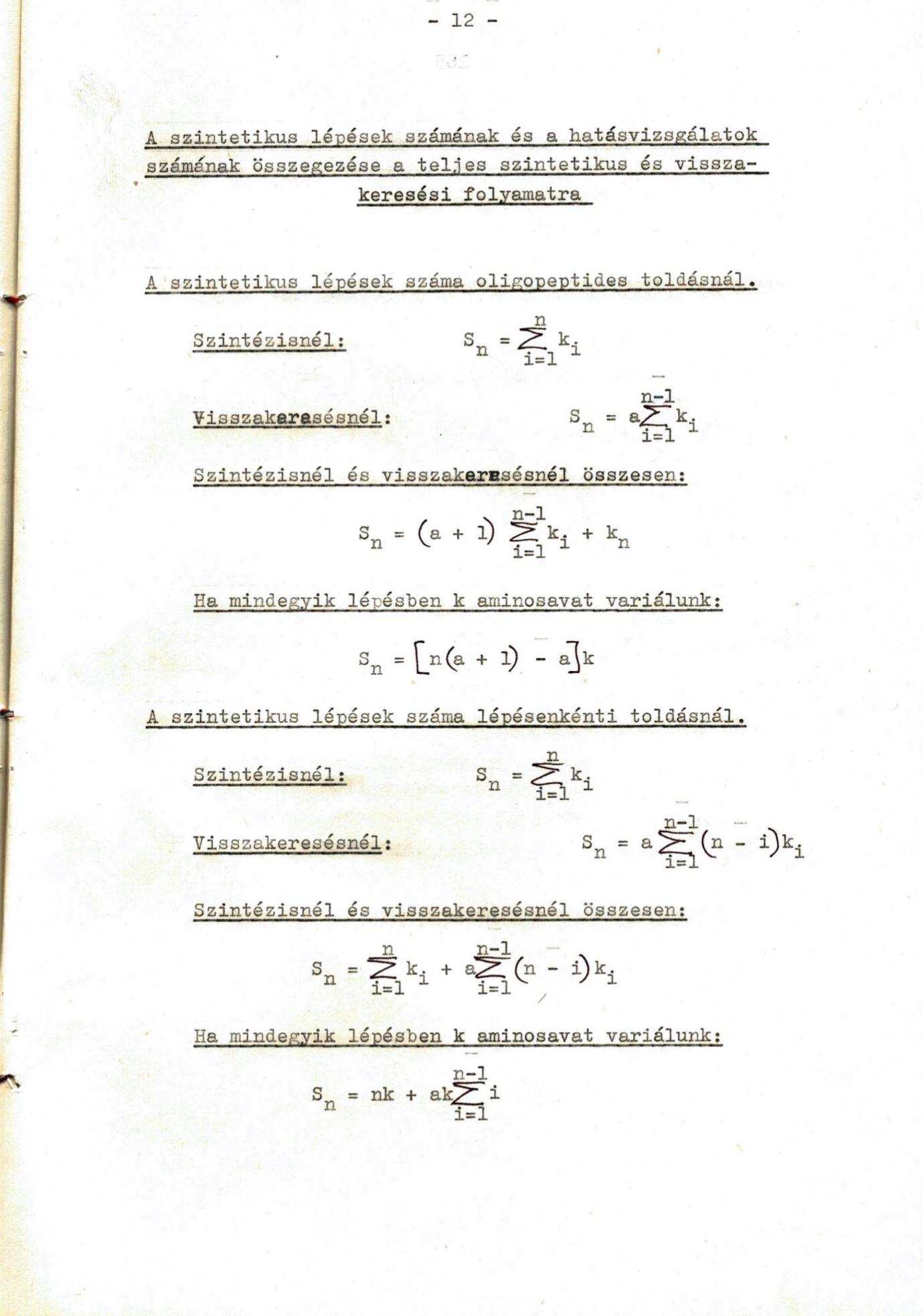


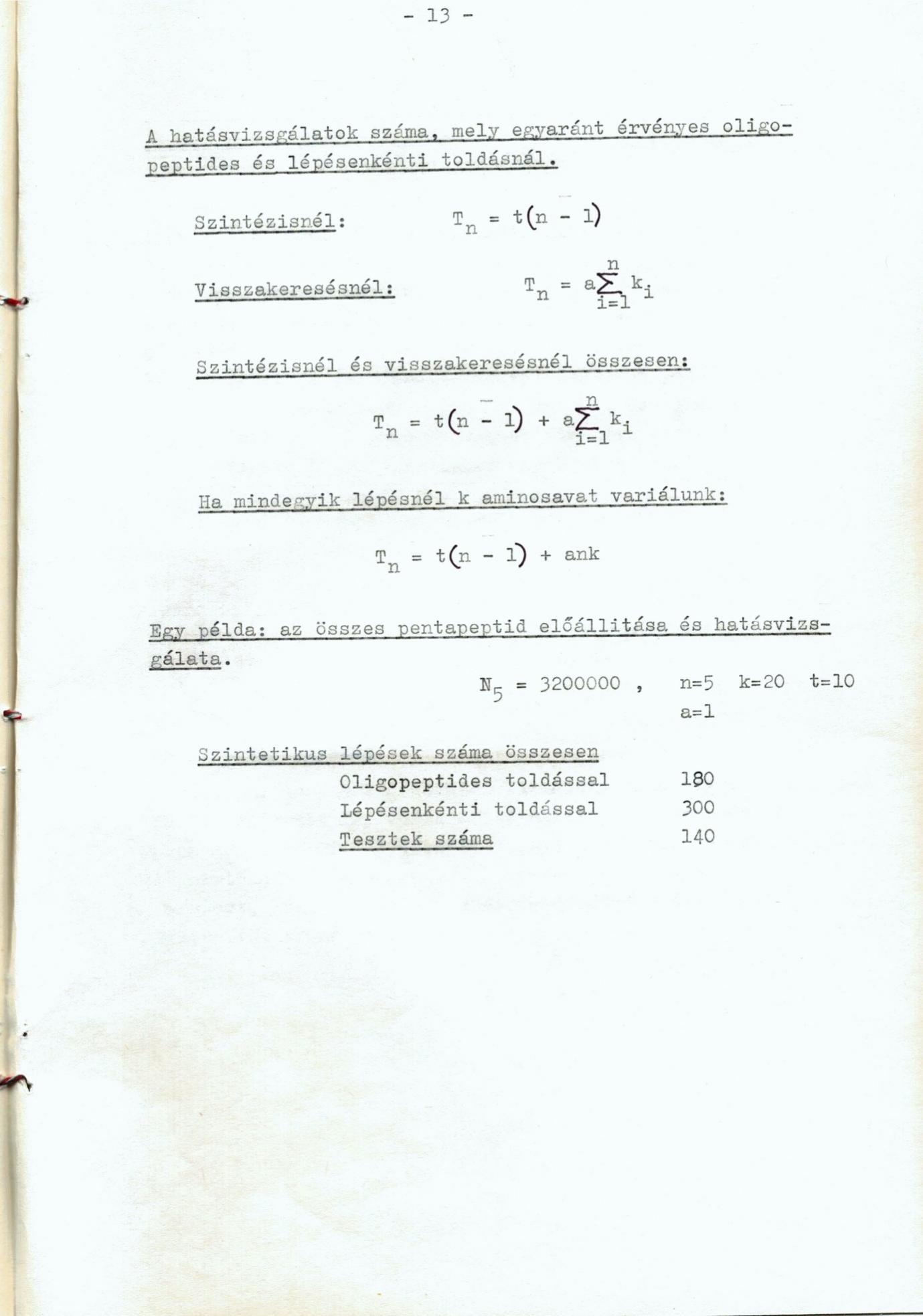


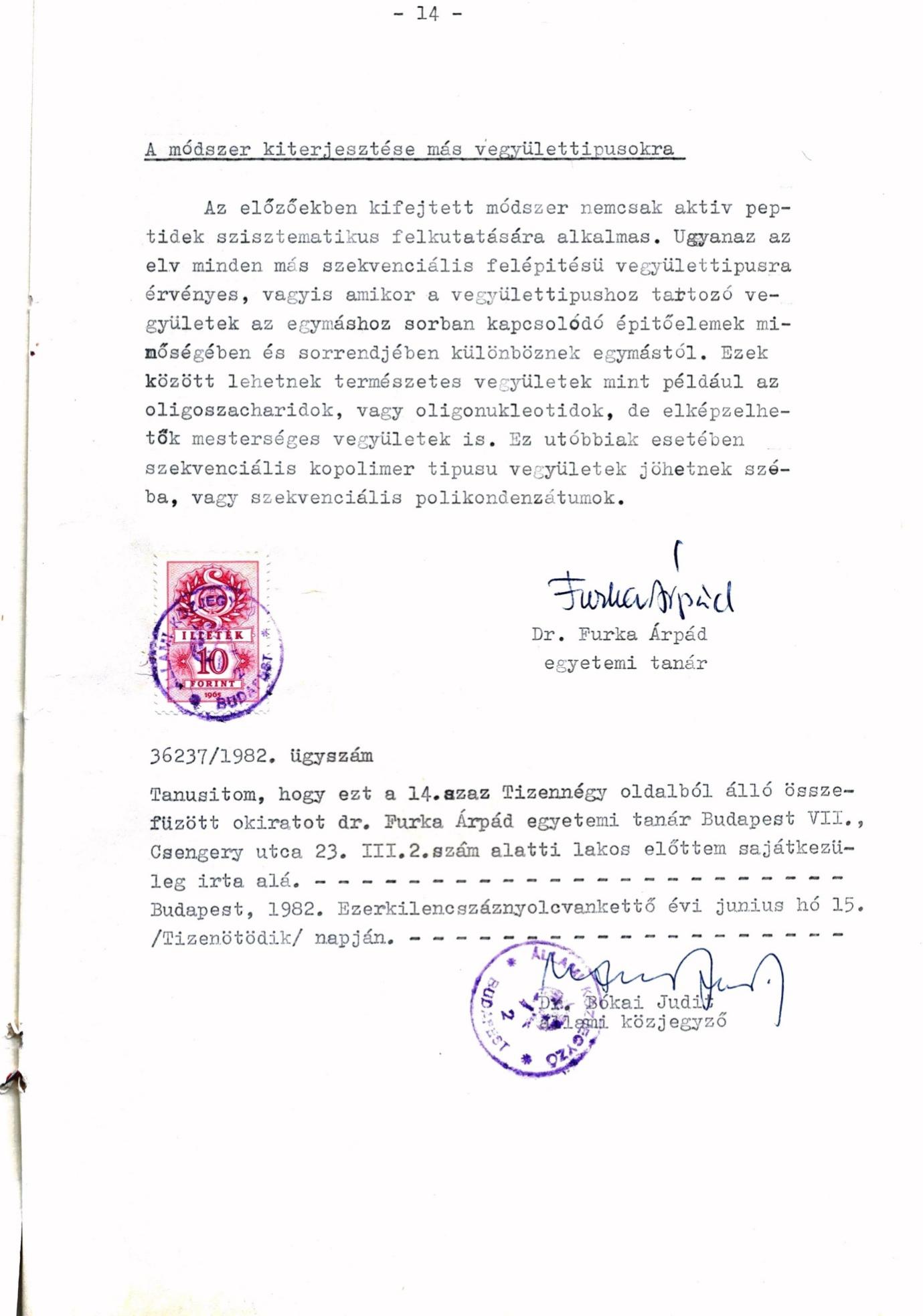


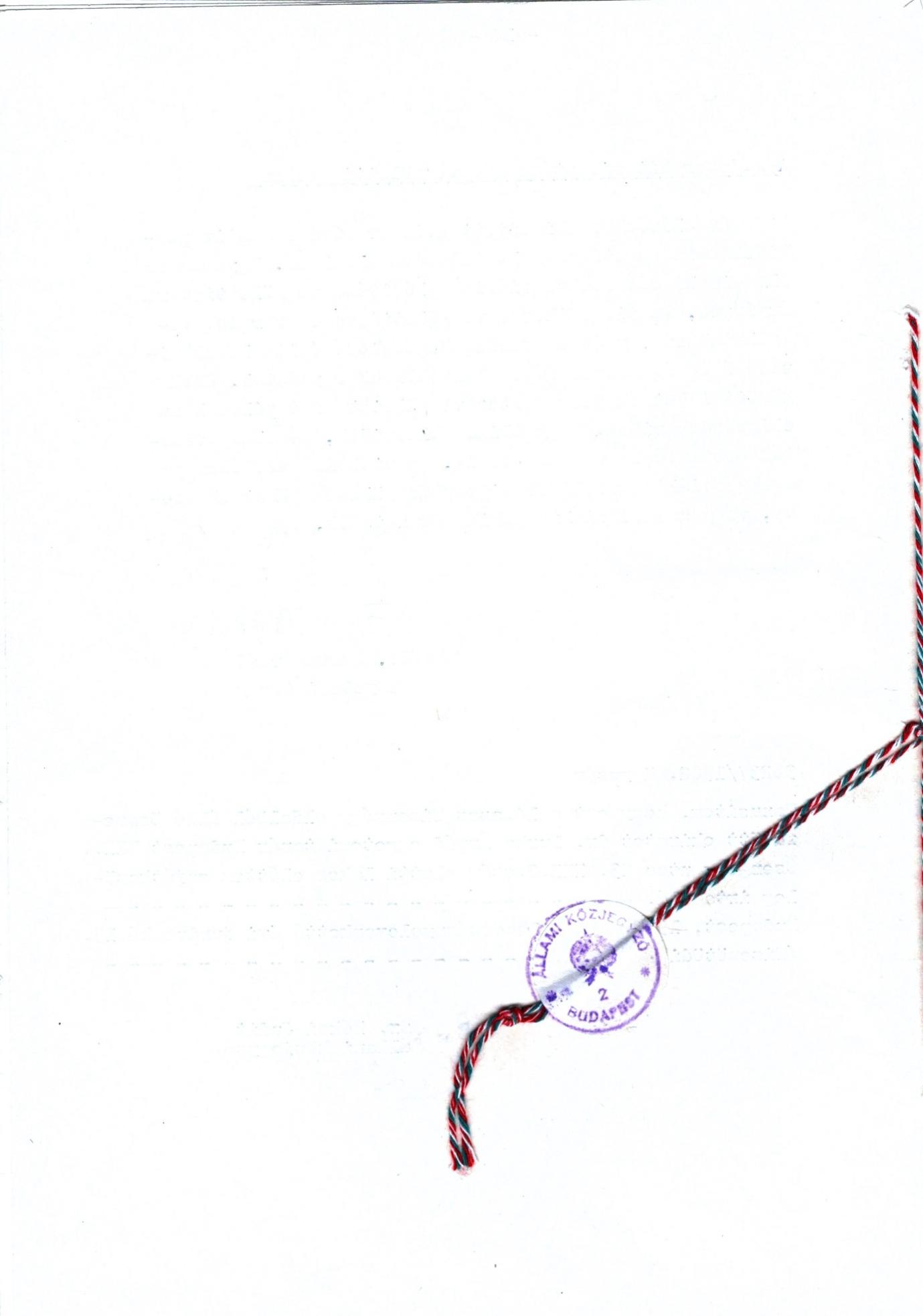


**The first description of the principles of combinatorial chemistry**

**Author: Professor Árpád Furka**

**Eötvös Loránd University Budapest Hungary**

**English version**

**The original Hungarian document was notarized in June 15, 1982**

STUDY ON POSSIBILITIES OF SYSTEMATIC SEARCHING FOR PHARMACEUTICALLY USEFUL PEPTIDES

Written by Dr. Árpád Furka, university professor

Budapest, May 29, 1982

As exemplified, among others,  by the peptide hormones discovered so far, the shorter-lenghtier peptides take part in a number of important functions in the living organism. It can be supposed, that only a small fraction is known of these biologically active peptides having potential therapeutic effect. This fact motivates the intensive international and domestic research activity in this field.
        Two, in principle different, approaches offer themselves for searching for peptides bearing new biological effects:

1. Isolation of peptides from living organisms based on their previously known biological effects.
2. Preparation of peptides by synthesis with post determination of their biological effects.

        Until now the isolation procedure proved to be more effective in spite of the fact that this method is also very laborious. This may be explained by the fact that the number of possible peptides grows rapidly with the number of residues so even the synthesis of all tetrapeptides (160 thousands) seems to be a hopeless task. If we consider the 20 natural amino acids the dependence of the number (*N_n_*) of possible peptides on the number of residues (*n*) is expressed by the following formula:

*N_n_* = 20*^n^*

If the *n*-residue peptides are synthesized stepwisely and independently, the number of the required synthetic steps (*S_n_*) can be calculated as follows:

*S_n_* = (*n*-1) 20*n*

 It is noted, that a synthetic step means a complete coupling cycle, that is, in addition to the coupling step itself incorporates the operations connected with the protecting groups, too.
        With good organization, that is, choosing a systematic synthesis route the number of synthetic steps can be reduced. The minimum number of synthetic steps is:

$$S_{n}=\sum_{i=2}^{n} 20^{i}$$

        The synthesized peptides are supposed to be submitted to screening tests. Since several tests have to be done on each peptide, the total number of the required screening tests is hopelessly large. If the number of kinds of screening tests is denoted by *t*, the total number of screening tests is expressed by the following equation:

*T_n_*  = *t* 20*^n^*

        Table 1 shows the the possible number of peptides depending on the number of residues, the number of synthetic steps required for their synthesis, and number of the screening tests, calculating with 10 different tests (*t*=10). The figures - which are rounded - clearly show, that even the synthesis and testing of all tripeptides would be an almost hopeless venture.

**Table 1**

Possible number of peptides (*N_n_* ) containing different number of residues (*n*),

the number of synthetic steps required for their synthesis (*S_n_* ) in an optimized

process, furthermore the number of screening experiments (*T_n_* ) calculating

with 10 different screening tests (*t*=10)

(the figures are rounded)

| *n* |  | *N_n_* | *S_n_* | | *T_n_* | |
| --- | --- | --- | --- | --- | --- | --- |
| 2 | 4 | hundred | 4 | hundred | 4 | thousand |
| 3 | 8 | thousand | 8 | thousand | 80 | thousand |
| 4 | 160 | thousand | 168 | thousand | 2 | million |
| 5 | 3 | million | 3 | million | 30 | million |
| 6 | 64 | million | 67 | million | 640 | million |
| 7 | 1 | billion | 1 | billion | 13 | billion |
| 8 | 25 | billion | 26 | billion | 256 | billion |
| 9 | 512 | billion | 537 | billion | 5 | trillion |
| 10 | 10 | trillion | 10 | trillion | 102 | trillion |

    Because of the very large number of possible peptides, the stepwise synthesis of all peptides - even in the case of small ones - is an unrealizable task. The large number of the screening experiments constitutes a further problem. The proposal to be outlined on the next pages will try to somewhat improve this almost hopeless situation.

Systematic search for biologically active small peptides through synthesis and screening of peptide mixtures

The proposal to be outlined here constitutes a research project which makes possible to search for biologically active peptides with much greater chance than before. When I write down this project I'm fully aware of its potential importance in industry. It is also clear, that it's realization is possible only through cooperation of different institutions. Primarily the participation of the pharmaceutical industry is desirable since the investments can be recovered through pharmaceutical industry.
  The essence of the proposal is that instead of one by one synthesis of peptides, peptide mixtures should be prepared containing several hundred or several thousand peptides in approximately 1 to 1 molar ratio, and these peptide mixtures should be submitted to screening tests. It will be shown that on this way much labor can be saved both in the synthetic work and in the screening experiments. In the first stage one has to determine whether or not the mixture shows any biological effect. If biological effect is observed, of course, it has to be determined which component (or which components) are responsible for the activity.

## Method for synthesis of peptide mixtures

Since not single peptides but rather mixtures of peptides are synthesized, post synthetic purification and removal of by-products are out of question. Because of this, the classical method of synthesis (in solution) can not be used either. In the synthesis of peptide mixtures the solid phase method have to be applied. It is noted here, that in the syntheses not necessarily the 20 amino acids are used. In some cases more than 20 amino acids may be used, for example if - in addition - non-common amino acids are intended to be used as building blocks. Less than 20 amino acids may be used, for example, in decapeptides, since the synthesis of all peptides seems to be unrealistic and have to compromise with the use of fewer kinds of amino acids. Let denote by *k*  the number of the amino acids intended to vary in the *i*  position. The numbers of amino acids varied in the C-terminal and N-terminal position are *k*_1_ and *k_n_* , respectively.

*Realization of the synthesis*

The resin is divided into *k*_1_  equal portions (that is to as many portions as many amino acids are intended to vary at the C-terminal of peptides). Then each portion of resin is coupled with one of the *k*_1_  kinds of amino acids then the amino-protecting group is removed from every sample. A small quantity is removed from every sample and they are taken aside for later use, then the samples are thoroughly mixed. Then the mixture of aminoacyl resins is divided into *k*_2_  equal portions and each of them is coupled with one of the *k*_2_  kinds of protected amino acids then the amino-protecting groups are removed from each sample. Before mixing, again small samples are removed and taken aside. The mixture of dipeptides is cleaved from a small portion of the mixed resin to use it in biological tests. The rest of the mixed resin is divided into *k*_3_  equal parts and the amino acids intended to occupy the third position are coupled to them. Then the synthesis is likewise continued until the mixture of *n*-residue peptides is reached.
        It is worthwhile to add some notes. As in an ordinary solid phase synthesis, one has to make an effort to achieve good conversion by applying the reagents in excess. Fortunately, however, conversions lower than 100%, or minor unwanted splitting reactions do not cause so serious problems like in ordinary syntheses. The labour requirement could be significantly reduced by using mixtures of properly protected amino acids in acylation reactions. This, however, does not seem to be an acceptable solution because of the differences in the reactivity of the activated amino acids which would lead to the formation of peptides in significantly different concentrations thus causing problems in the screening experiments. Formation of peptides in equal concentrations can only be assured by mechanical mixing of samples followed by dividing into equal portions. This makes possible a complete conversion for every amino acid component. Possibility of acylations with mixtures of several amino acids of identical reactivity might be a matter of further considerations. Smaller differences in reactivities could be compensated by properly selected molar ratios of the amino acid derivatives of the mixture. In the following calculations the possibility of acylations with the mixtures of amino acid derivatives will be left out of considerations.
        The number of peptides formed in the synthesis, that is, the number of components in the peptide mixtures - in a general case - can be calculated by the following formula:

*N_n_* = *k*_1_.*k*_2_ . . . . . . *k_n_*_-1_.*k_n_*

If the same number (*k*) of amino acids are varied in every position

*N_n_ = k^n^*

The number of synthetic steps in the synthesis of a peptide mixture containing *N_n_*  peptides (considering the attachment of the first amino acid to the resin as separate step) is:

*S_n_* = *k*_1_ + *k*_2_ + . . . . + *k*_n-1_ + *k_n_*

If the same number (*k*) of amino acids are varied in each position,

*S_n_* = *nk*

The formulae show the advantage of the synthesis of peptide mixtures: the number of the synthetic steps can be calculated by summing the numbers of the varied amino acids, while the number peptides is given by the product of the numbers of the varied amino acids.
        One example: synthesis of the mixture of tetrapeptides prepared by varying the 20 kinds of amino acids, needs only 80 synthetic steps! It is noted, that in the same run all shorter peptides - that is the 400 dipeptides and the 8000 tripeptides - are formed, too. The traditional synthesis of these peptides would need 168 400 synthetic steps. A different comparison: in the traditional method with 80 steps only about 30 tetrapeptides can be synthesized.

**Screening of peptide mixtures**

Peptides mixtures - in the first approximation - are synthesized to determine whether or not they contain biologically active component. It is supposed - although it needs experimental verification - that screening experiments can be made with mixtures, too. This offers great advantage over the traditional method since the number of screening tests is reduced by a factor equal to the number of components of the mixture. For example, the mixture of the 8000 tripeptides can be examined by a single series of tests. If there is active peptide among them, one of the executable *t* tests gives positive result. If the number of active peptides is more than one, then, of course, more tests may give positive result. In the synthesis of the mixture of *n*-residue peptides it is wortwhile to test the shorter peptides, too. The synthesis is so designed to allow for this. Taking this requirement into account, and the number of kinds of tests being *t*, the total number of the executable tests is:

*T_n_* = *t*(*n*-1)

Although this equation certainly holds, its realizability in practice deserves some notes. There is - without any doubt - an upper limit in the number of components of the peptide mixtures to be submitted to screening tests. It is difficult to estimate this number without experiments. The mixtures may probably contain many thousands of components, and as it can be judged today, the method outlined above is rather limited by possibilities of screening tests than by the number of the required synthetic steps. If there are too many components in the mixture, too large samples have to be applied in the screening experiments to achieve observable effect for a single component. The mixture supposedly contains a number of more or less active analogs and their effect is probably summarized. Nevertheless, an unsurpassable limit in the number of components certainly exists. Therefore in certain cases may prove useful to examine the effect of the *n*-residue mixtures without final mixing. In other cases the synthesis should be designed so not to surpass the optimal number of components.

*"Backsearching" for the active peptide*

If the peptide mixture is detected to contain active component, that is, if the mixture shows a new type biological effect, then the further task is the isolation and structure determination of the active peptide followed by its synthesis. Once the mixture containing the active component or components is in our hand the isolation can be carried out using the effective separation methods, since these make possible to separate the active compound even from thousands of inactive components. It is possible, however, to follow a different method, too. This will be outlined here. This approach to the identification of the active peptides is supposed to be less tedious then the isolation method, moreover it supplies additional information concerning the structure-effect relationship. Applicability of the method requires a procedure for quantitative determination of activity. For the sake of simplicity let's suppose that the mixture contains a single effective component (besides analogs having the same kind of effect but smaller activity).

*Backsearching step No. 1*

The experiments are started with the *k_n_* samples taken aside in the synthesis of the *n*-residue peptides before final mixing. The mixtures of *n*-residue peptides are cleaved from each resin sample. The mixtures of peptides differ from each other in the *n-*th   (that is the N-terminal) residue of their component peptides. Each peptide mixture is submitted to a quantitative activity determination. This shows how the activity depends on the terminal amino acid residue, that is, this way we can determine the N-terminal residue of the active peptide, and in addition it will show the effect of its replacement by other amino acid residues. Let's suppose, for example, that the N-terminal residue in the sample showing the highest activity (as well as in the active peptide) is Phe (phenylalanine). It is noted here that if there are several samples showing equally high activity it is practical to choose as the N-terminal residue of the active peptide the cheapest or the synthetically less problematical amino acid. This note holds for the subsequent backsearching steps, too.

*Backsearching step No. 2*

The experiment is continued with the *k_n_*_-1_ samples taken aside in the synthetic stage of the (*n*-1)-residue peptides. The amino acid determined before, that is Phe in our example, is coupled to each sample. Cleavage of the peptides from the support gives *k_n_*_-1_ different peptide mixtures. Their common feature is that every peptide has Phe in the N-terminal position. By submitting the peptide mixtures to quantitative screening experiments one can determine the amino acid residue occupying position *n*-1 (that is, the pre-N-terminal position) in the active peptide. This experiment also shows the effect on activity of substitution of this amino acids with other ones. Let's suppose that the pre-aminoterminal amino acid is Arg (arginine). It should be noted that in this backsearching step the Phe is coupled to *k_n_*_‑1_ samples and the same number (*k_n_*_-1_) of screening experiments have to be done. Not all of the *t* kinds of tests are required, only the one proved before to be positive. Consequently the number of the synthetic steps and the number of screening experiments are the same: *k_n_*_-1_. It is also noted that in the previous backsearching step only screening test are done (their number is *k_n_*) synthetic steps are not needed.

*Backsearching step No. 3*

This, and the subsequent backsearching steps may be realized using two different approaches. The peptides in samples taken aside during the synthesis have to be elongated to contain *n* residues, in such way, to carry on their N-terminal section the amino acid residues assuring activity. This can be realized on two ways. Either by stepwise coupling with amino acids (in our example with protected Arg then Phe) or by coupling in a single step with a previously synthesized oligopeptide having the required sequence (in our example Phe.Arg). The required synthetic steps in the two approaches significantly differ. The number of the screening experiments, however, are the same in both cases. Let's turn now to the No. 3. backsearching step.

*Stepwise elongation*

Let's take the *k_n_*_-2_ samples taken aside in the synthesis of (*n*-2)-residue peptides. Each sample is coupled first with protected Arg then with protected Phe. After cleaving the peptides from the support each of the *k_n_*_-2_ peptide mixtures are submitted to activity tests to determine the amino acid residue occupying the third position counting from the N-terminal end. The number of screening tests to be executed is *k_n_*_-2_. The number of the required synthetic steps is: 2*k_n_*_-2_. The multiplying factor preceding *k*  is the bigger the shorter are the peptides to be elongated. The numerical value of the factor is equal to the number of amino acids to be coupled with in the elongation process.

*Elongation with oligopeptide*

A previously synthesized dipeptide (in our example Phe.Arg) is coupled to each of the *k_n_*_-2_ samples taken aside, then the process is continued as described above. The number of screening test is also *k_n_*_-2_. The number of synthetic steps (leaving out of consideration the synthesis of the oligopeptide) is also *k_n_*_-2_. This procedure seems to be more economical. In practice it means that the active peptide is synthesized in parallel with the screening tests using the classical method started from the N-terminus. Small fractions of the growing peptide are sacrificed in the backsearching steps. This backsearching method has the great advantage (in addition to the fact that it needs less synthetic steps) that when the backsearching procedure is finished the active peptide is synthesized, too.

*Backsearching of more than one active peptide*

In the synthetic peptide mixtures several active peptides may be present, showing different effects. In these cases the number of backsearching steps will be bigger by a factor equal to the number of the differing active peptides. That is, if the number of the active peptides is "*a*" the values deduced above are multiplied by *a*. It is noted that the presence in the mixture of peptides having different effects may complicate the backsearching process especially in the case of peptides with opposing effects. This, however, is not treated in details.
        The backsearching process ends when the sequences of all active peptides are determined by applying either the oligopeptide or the stepwise elongation method.

**Total number of synthetic steps and screening tests summarized**

**for the whole synthetic backsearching process**

*Number of synthetic steps using oligopeptide elongation*

         In synthesis:                                                   $S_{n}=\sum_{i=1}^{n} k_{i}$

         In backsearching:                                         $S_{n}=a\sum_{i=1}^{n-1} k_{i}$
         Total in synthesis and backsearching:         $S_{n}=(a+1)\sum_{i=1}^{n-1} k_{i}+k{}_{n}$

         If k amino acids are varied in each step:        *S_n_* = [*n*(*a* + 1) - *a*]*k*

*Number of synthetic steps using stepwise elongation.*

         In synthesis:                                                    $S_{n}=\sum_{i=1}^{n} k_{i}$

         In backsearching:                                           $S_{n}=a\sum_{i=1}^{n-1} (n-1)k_{i}$

         Total in synthesis and backsearching:            $S_{n}=\sum_{i=1}^{n} k_{i}+a\sum_{i=1}^{n-1} (n-1)k_{i}$

         If k amino acids are varied in each step:        $S_{n}=nk+ak\sum_{i=1}^{n-1} i$

*Number of screening tests equally valid using the oligopeptide and stepwise elongation*

         In synthesis:                                                    *T_n_* = *t*(*n*-1)

         In backsearching:                                           $T_{n}=a\sum_{i=1}^{n} k_{i}$

         Total in synthesis and backsearching:          $T_{n}=t(n-1)+a\sum_{i=1}^{n} k_{i}$

         If *k* amino acids are varied in each step:         *T_n_* = *t*(*n*-1) + *ank*

*An example: preparation and screening of all pentapeptides*

*N*_5_ = 320000,      *n*=5     *k*=20     *t*=10     *a*=1

         Total number of synthetic steps

                                               Ogopeptide elongation          180
                                               Stepwise elongation              300
                                               Number of tests                    140

**Extension of the method to other types of compounds**

Applicability of the method outlined before is not restricted for only the systematic searching for active peptides. The same principle applies to all other sequential types of compounds, that is, when the compounds belonging to this type of compounds differ from each other only in their building blocks or the sequences of these building blocks. Among them may occur natural compounds like oligosaccharides or oligonucleotides but synthetic products may be taken into account, too. Among these later ones one may think about sequential copolymers or sequential polycondensates.

                                                                                                               Dr. Arpad Furka
                                                                                                               university professor
 

File number 36237/1982
I certify this stitched document comprising 14, that is, fourteen pages was subscribed in my presence by Dr. Arpad Furka, university professor, with his own hands.
Budapest, 1982. Nineteen hundred and eighty two, June 15, (fifteen).
                                                                                                                 Dr. Judit Bokai
                                                                                                                 state notary public

**OBOC libraries and deconvolution methods**

**Formation of OBOC libraries**

It is an intrinsic feature of the split and pool synthetic method that a single compound forms on each bead. The structure of the compound depends on the reaction vessels in which the bead happens to occur in the synthetic route.


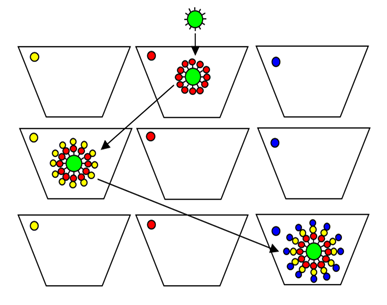


Figure 1. Formation of OBOC libraries in the split and pool santhesis

**Recursive deconvolution**

The method is made understandable by Figure. A 27 member peptide library is synthesized from three amino acids. After the first (A) and second (B) cycles samples were taken aside before mixing them. The products of the third cycle (C) are cleaved down before mixing then tested for activity. Suppose the group labeled by + sign is active. All members have the red amino acid at the last coupling position (CP). Consequently, the active member also has the red amino acid at the last CP. Then the red amino acid is coupled to the three samples taken after the second cycle (B) to get samples (D). After cleaving the three (E) samples are formed. If after testing the sample marked by + is the active one it shows that the blue amino acid occupies the second coupling position in the active component. Then to the three (A) samples first the blue then the red amino acid is coupled (F) then tested again after cleaving (G). If the + component proves to be active, the sequence of the active component is determined and shown in (H).

A

B

 C

**+**

**+**

D

E

F

G

H

**+**

Figure 2. Blue, yellow and red circles: amino acids, Green circle: solid support.

**Positional scanning**

The method is explained in the Figure. The three columns on the left side of the Figure show a 27 member trimer library built from three amino acids. The nine sub-libraries are shown on the right side. In all sub-libraries, there is a coupling position (CP) that is occupied by a single amino acid. In the remaining CPs all three amino acids are varied. If the upper left sub-library shows activity it means that the red amino acid occupies the CP 1 in the active peptide. Similarly, the result of testing all the sub-libraries identifies the sequence.

3 2 1 3 2 1 3 2 1

1

2

3

1

2

3

1

2

3

Figure 3. Blue, yellow and red circles: amino acids. The numbers show coupling positions.

**Omission libraries**

Omission libraries are prepared by omitting a certain amino acid in all coupling positions in the synthesis of the library (see them in the 2-3 columns). As a consequence, a certain amino acid is missing from all peptides of the mixture. If the omission library gives a negative test the omitted amino acid is present in the active component The Figure shows an example. The “blue” and the “red” omission library shows the negative results and this means that the “blue” and the “red amino acid is present in the bioactive peptide.


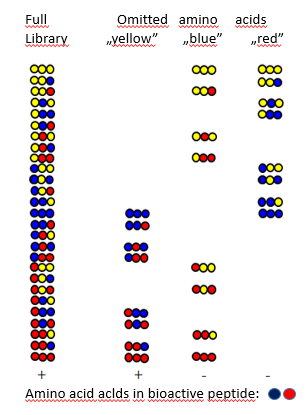


Figure 4.

**Demonstration of the applicability of the above described methods**

Árpád Furka: unpublished results

Applicability of the screening strategies described before is demonstrated by a few model experiments. The task in these experiments was to determine whether or not a synthesized tripeptide library has a component that inhibits the binding of LHRH^14^ to its antibody. The amino acid sequence^15^ of the hormone is shown below:

pGlu-His-Trp-Ser-Tyr-Gly-Leu-Arg-Pro-Gly-NH2

The LHRH polyclonal antibody, as well as the radioactively labeled LHRH, were the products of Advanced ChemTech. The competitive inhibition of LHRH to its antibody was determined by radioimmunoassay^16^ (RIA).

**Testing the full tripeptide library**. Since the LHRH is a decapeptide amide, a tripeptide amide library was prepared and used in screening. In the solid phase split-mix synthesis of the tripeptide amide library 19 amino acids were used in the first and second coupling positions (cysteine was omitted) and, because LHRH has pyroglutamic acid at the N-terminal position, pyroglutamic acid was added to this set in coupling position 3. The library was prepared on Rink amide resin using the F-moc strategy and the automatic split and pool synthesizer of AdvancedChemTech (ACT).


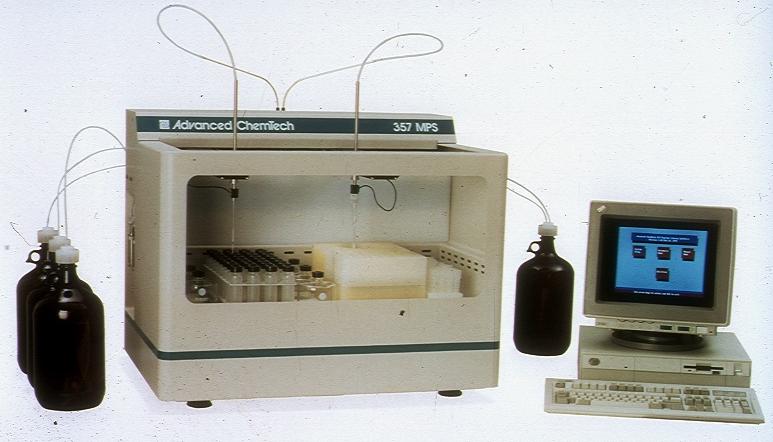
.

Figure 5. The ACT 357 automatic split-mix synthesizer

The tripeptide amide library was added in increasing concentrations to the mixture of radioactive LHRH and its antibody and their binding was determined by RIA. The result is demonstrated in Figure 6. It can be seen that binding is strongly reduced by increasing the concentration of the library. This makes it probable that the library has component/s that inhibit binding, that is, it is worthwhile to make further experiments in order to identify this component. The result also suggests that the optimal concentration for the binding experiments should be around 50 microgram/ml. In all the further experiments the libraries were applied in molarities equivalent to this concentration.


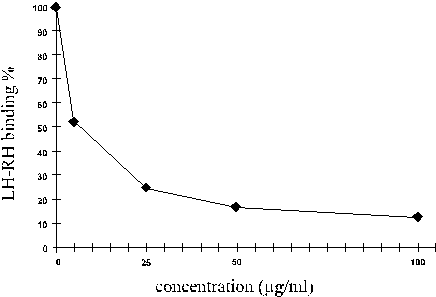


Figure 6. Effect of concentration of the full tripeptide library

on binding of LHRH to its antibody

**Recursive deconvolution. The first step**. Before the final mixing in the split-mix synthesis of the full tripeptide amide library, equal samples were removed and cleaved from the support. These mixtures were suitable to demonstrate the first step in the iteration strategy.

Figure 7. Inhibitory effect of sub-libraries used in the first iteration step. 3p denotes pyroglutamic acid in the N-terminal position

Figure 7. shows how strong is the inhibitory effect of the sub-libraries. It can be clearly seen that sub-library 3R exhibits the far strongest effect. This means that the amino acid occupying the coupling position 3 in the inhibitory tripeptide amide is arginine, R.

**Application of omission libraries.** Omission libraries were derived from the tripeptide amide full library described above. Thus in the synthesis of 19 omission libraries (-A to –Y) one amino acid was omitted in all three coupling positions. The remaining 18 amino acids were built-in into all positions. Pyroglutamic acid was also present in all omission libraries in coupling position 3.

Pyroglutamic acid omission library could not be prepared since this amino acid can be inserted only into coupling position 3 of the tripeptides. It was also important, however, to test whether or not this amino acid is present in the active peptide. For this reason a full tripeptide amide library was prepared by using 19 amino acids in each coupling position and the pyroglutamic acid was omitted from position 3 (denoted by –p).

The importance in inhibition of the amide groups of the peptides was also tested. In order to do this one part of the full library of tripeptides was cleaved from the support in the form of carboxylic acids instead of amides and tested in this form (denoted by -a).

When tested the omission libraries, the full tripeptide amide library (denoted by X) was also included. The result is demonstrated in Figure 8. It can be seen that the omission libraries that less reduces the competitive binding are: -G, -P, -R, and –a. This means that the amino acid composition of the inhibitory tripeptide is glycine (G), proline (P), and arginine R. The peptides that do not have amide groups are not effective inhibitors. This means that the amide group is also an essential part of the inhibitory tripeptide.

Figure 8. Effect of omission libraries on binding.

X and -a mean full tripeptide amide and full tripeptide acid libraries, respectively,

while -p denotes the library from which pyroglutamic acid was omitted. The other omission

libraries are represented by a minus sign followed by the one-letter symbol of the

omitted amino acid

The results of the experiments carried out with omission libraries gave no indication about the position of the amino acids within the sequence of the active peptide. Despite this, the information gained by only 21 screening experiments is very valuable. They define an „amino acid occurrence library” that can be synthesized by varying only three amino acids, Gly, Pro, and Arg in all of the three coupling positions. The inhibitory tripeptide is expected to be present among the 27 components of this tripeptide amide library. In other words, by screening with omission libraries, the complexity of the library in which the active peptide is found could be reduced from the original 7220 to only 27.

The positions of the identified amino acids could be determined by using one of the following three possibilities:

1. Preparation by parallel synthesis and screening of the 27 components of the occurrence library.
2. Application of positional scanning to the occurrence library (preparation and screening of nine sub-libraries).
3. Positional scanning with nine sub-libraries of the full library (if available).

**Synthesis and use of positional scanning libraries**. Screening with omission libraries showed that binding of LHRH to its antibody is inhibited by a tripeptide amide having composition glycine, proline, and arginine. As outlined before, based on this result an occurrence library can be defined. If this library is synthesized, it contains among its components the inhibitor tripeptide amide (Table 1.).

Table 1. Building blocks of the occurrence library

| Coupling posirion | Amino acids | | |
| --- | --- | --- | --- |
| 1 | G | P | R |
| 2 | G | P | R |
| 3 | G | P | R |

The position of the three amino acids in the sequence of the inhibitor tripeptide amide is determined by synthesizing and testing of the nine component kit of the positional scanning library. The synthesis is optimized to make it possible to prepare the nine component libraries of the kit (1G, 2G, 3G, 1P, 2P, 3P, 1R, 2R and 3R) in a single run on the automatic synthesizer ACT 357. The solid support was again Rink resin. The synthesizer was pre-programmed that made possible to execute the whole process automatically.

Coupling with G

Coupling with P

Coupling with R

Mix

Split

Mix & Split

2G 2P 2R 1G 1P 1R

3G 3P 3R

Start

1/3 1/3 1/3

1/2 1/2 1/2

Figure 9. Flow diagram of the synthesis of the 9 positional scanning sub-libraries

of the occurrence library

Figure 9

The flow diagram is demonstrated in Figure 9. The starting resin, placed into the collection vessel was first divided into three portions then coupled with glycine, proline, and arginine, respectively. Before mixing, 1/3 part of each reaction product was transferred into a separate reaction vessel then each of them was individually submitted to two consecutive portioning-mixing cycles coupling in each cycle with glycine, proline, and arginine, yielding 1G, 1P, and 1R as the end product. The remainder was mixed, divided into three parts then each part coupled with one of the three amino acids. Again, before mixing, 1/2 part of each sample was transferred to a separate reaction vessel then individually submitted to a full portioning mixing cycle, using again glycine, proline, and arginine in couplings. These operations resulted in the formation of 2G, 2P, and 2R. The remainder was mixed, divided into three portions then each coupled with one of the three amino acids. The three products were 3G, 3P, and 3R.

The synthesized nine first-order sub-libraries were used to determine the position of glycine, proline, and arginine in the tripeptide responsible for competitive inhibition of binding of LH-RH to its antibody (Figure 10).

Figure 10. Positional scanning by sub-libraries of the occurrence library

Since on the y axis not LHRH binding% but instead 100-LHRH binding% is plotted, Figure 10. shows that the inhibition of binding of LHRH to its antibody is strongest in the case of the 1G, 2P, and 3R sub-libraries. Consequently, arginine, proline, and glycine occupy the coupling positions 3, 2, and 1 in the tripeptide, respectively. The sequence of the inhibitor tripeptide is Arg-Pro-Gly-NH_2_. This sequence happens to be identical to the C-terminal sequence of LHRH.
